# CircCDK14 protects against Osteoarthritis by sponging miR-125a-5p and promoting the expression of Smad2

**DOI:** 10.7150/thno.45993

**Published:** 2020-07-11

**Authors:** Panyang Shen, Yute Yang, Gang Liu, Weijie Chen, Junxing Chen, Qingxin Wang, Hongliang Gao, Shunwu Fan, Shuying Shen, Xing Zhao

**Affiliations:** 1 Department of Orthopaedic Surgery, Sir Run Run Shaw Hospital, Zhejiang University School of Medicine.; 2 Key Laboratory of Musculoskeletal System Degeneration and Regeneration Translational Research of Zhejiang Province.; 3 The Hospital of the Marine Police Corps of the Chinese people's Armed Police Force.; 4 Departments of Orthopedics, Huzhou Central Hospital, Huzhou City, Zhejiang Province.

**Keywords:** CircCDK14, Osteoarthritis, miR-125a-5p, Smad2, metabolism

## Abstract

**Rationale:** Osteoarthritis (OA) is the most common joint disease worldwide. Previous studies have identified the imbalance between extracellular matrix (ECM) catabolism and anabolism in cartilage tissue as the main cause. To date, there is no cure for OA despite a few symptomatic treatments. This study aimed to investigate the role of CircCDK14, a novel circRNA factor, in the progression of OA, and to elucidate its underlying molecular mechanisms.

**Methods:** The function of CircCDK14 in OA, as well as the interaction between CircCDK14 and its downstream target (miR-125a-5p) and mRNA target (Smad2), was evaluated by western blot (WB), immunofluorescence (IF), RNA immunoprecipitation (RIP), quantitative RT-PCR, luciferase assay and fluorescence *in situ* hybridization (FISH). Rabbit models were introduced to examine the function and mechanism of CircCDK14 in OA *in vivo*.

**Results:** In our present study, we found that CircCDK14, while being down-regulated in the joint wearing position, regulated metabolism, inhibited apoptosis and promoted proliferation in the cartilage. Mechanically, the protective effect of CircCDK14 was mediated by miR-125a-5p sponging, which downregulated the Smad2 expression and led to the dysfunction of TGF-β signaling pathway. Intra-articular injection of adeno-associated virus-CircCDK14 also alleviated OA in the rabbit model.

**Conclusion:** Our study revealed an important role of CircCDK14/miR-125a-5p/Smad2 axis in OA progression and provided a potential molecular therapeutic strategy for the treatment of OA.

## Introduction

Osteoarthritis (OA), a chronic degenerative joint disease that is most commonly in the elderly, is the leading cause of disability worldwide 1. It is characterized by the failure of cartilage repair processes to compensate for cartilage injury 2. Subchondral bone sclerosis, osteophyte formation and synovial hyperplasia also play pivotal roles in the development of OA 3-5. A large number of risk factors have been shown to be associated with the progression of OA, including aging, gender, obesity, genetics, joint laxity, muscle weakness and previous joint injury 6-8. Despite the growing economic and societal impact of OA 9, an in-depth understanding of its pathogenesis remains elusive. This explains the lack of effective treatment options for OA that take individual variability into account. Therefore, a deeper understanding of both its pathogenesis and treatment is urgently needed.

Micro ribonucleic acids (microRNAs) are non-coding single-stranded RNAs that are involved in a variety of inflammation-mediated diseases 10. Previous studies have confirmed the role of miRNAs in the progression of OA 11-14, including studies of metabolic processes, chondrocytes apoptosis and autophagy.

Circular ribonucleic acids (circRNAs) are characterized by a covalently closed-loop structure generated through backsplicing, a special type of alternative splicing 15, 16, and has been well documented to function in miRNA sponging 17. Considering the important role of miRNAs in OA development and the cross interaction between circRNAs and miRNAs, we hypothesize that circRNAs which enriched in miRNA-binding sites may also play a role in OA development by function as miRNA sponges. This is supported by previous studies which indicate that circRNAs could delay or aggravate the progression of OA. Shen at al. 18 reported that CircSERPINE2 protects against osteoarthritis by targeting miR-1271 and ETS-related gene. Zhou at al. 19 showed that circRNA.33186 directly binds to and inhibits miR-127-5p, thereby increasing MMP13 expression and contributes to OA pathogenesis. Therefore, circRNAs may be a novel molecular target for the treatment of OA.

The transforming growth factor β (TGF-β) signaling pathway, which is important in the transcription of multiple gene and normal cell function 20, 21, has been shown to be closely related to OA. The pathway can be divided into a Smad-dependent pathway and Smad-independent pathway 22-24. Smad proteins serve as a crucial signal transducer protein by which the TGF-β signals from the cell membrane to the nucleus 25, thereby mediating the more important Smad-dependent pathway.

In our present study, we identified a novel circRNA (CircCDK14) as a key protective factor in development of OA. Its protective effect was mediated by miR-125a-5p sponging, which downregulated the Smad2 expression, leading to the dysfunction of TGF-β signaling pathway.

## Methods

### Human tissue collection

Written informed consent was obtained from each patient and our research was approved by Ethics Committee of Sir Run Run Shaw Hospital (Hangzhou, China). Human cartilage samples were obtained from patients who underwent total knee replacement surgery for end-stage symptomatic knee (n=15). The degree of arthritis in patients was assigned according to the Outerbridge grade system 26. Patients with metabolic and autoimmune diseases, such as hypertension, hyperlipidemia, diabetes, rheumatoid arthritis and other diseases that affect joints, were excluded from this study. The area between the medial and lateral condyles of the distal femur serves as a non-weight-bearing area, while the medial and lateral tibial plateau serves as a weight-bearing area. Therefore, cartilage tissues from these two areas were collected for subsequent analysis. Since there was usually no cartilage wear in non-weight-bearing area, our assessment on the severity of arthritis was based on the degree of cartilage wear in the weight-bearing area (Figure S1).

### Animal models

Twenty-four male New Zealand white rabbits, aged 12 months, were used to introduce an OA model by anterior cruciate ligament transection (ACLT) as described in previous researches 27, 28. Briefly, in each case, the bilateral knee joint was entered through the medial capsule, and the anterior cruciate ligaments were exposed and transected. An anterior drawing test was performed to verify the success of the operation, the rabbit's knee was bended to 90 degrees, the femoral segment was fixed with the left hand, and a back-to-front force was applied through the tibia of the rabbit with the right hand. It was found that the tibia of the rabbit could be significantly displaced relative to the femur, indicating that its anterior cruciate ligament was completely broken. The joint was then closed following irrigation with sterile saline. Another group of eight male New Zealand white rabbits, aged 12 months, was used as the control group on which a sham operation was performed. The knee was opened, and the patella was dislocated. After performing the anterior test, the joint was irrigated and closed without transecting the anterior cruciate ligament. The c virus (AAV) CircCDK14 wt and negative control lentivirus were constructed and packaged by HanBio (Shanghai, China), the miR-UPTM agomir miR-125a-5p was designed and constructed by Genepharma (Shanghai, China). The rabbits were randomly divided into four groups (control group, NC injection, CircCDK14 injection and CircCDK14+miR-125a-5p injection) with eight rabbits in each group, and each rabbit was assigned a cage. One week after the ACLT surgery, a total of 100 µl solution containing above virus (approximately 1×10^8^ PFU/mL) and miR-UPTM agomir was slowly injected into right knee joint cavities without solution spill. All rabbits were sacrificed seven weeks after the injection, and the knee joints were cut out for immunohistochemistry. All animal experiments were performed with the approval from the Institute of Health Sciences Institutional Animal Care and Use Committee.

### AAV Construction

The c virus (AAV) CircCDK14 wt and negative control lentivirus (Sequences of CircCDK14 were inserted into XbaI restriction sites of pHBAAV-CMV-circRNA-EF1-ZsGreen vector) were constructed and packaged by HanBio (Shanghai, China). The vector pHBAAV-CMV-circRNA-EF1-ZsGreen was selected for use in this study, and restriction endonuclease XbaI (Thermo Scientific) was used for vector cleavage to obtain a purified linearized vector. CircCDK14 fragments were amplified using 2xFlash PCR MasterMix (Dye) kit (CWBIO) according to the manufacturer's instructions. An HB infusionTM kit (Hanbio Biotechnology) was then used for the ligation of the linearized vector and CircCDK14 fragments, according to the manufacturer's instructions, followed by transformation of DH5α competent cell (TIANGEN). After cultivating with LB medium in culture plates for 12-16 h, the bacterial solution was used for PCR identification with 2xHieff PCR Master MIX (Dye) kit (YEASON) according to the manufacturer's instructions and the amplified sequence was detected by Sanger Sequencing to verify the consistency with CircCDK14. The plasmid was extracted with TIANpure Mini Plasmid Kit (TIANGEN) according to the manufacturer's instructions. Finally, the extracted plasmid was co-transfected with Packaging plasmids (pAAV-RC and pHelper) into HEK-293T cells using LipofiterTM transfection reagent (HanBio) as per the manufacturer's instructions. After 72 h of transfection, the transfected HEK-293T cells were centrifuged and broken, and the supernatant was collected for virus purification using ViraTrap™ AAV Purification Maxiprep Kit (Biomiga). The purified virus samples were stored at -80 °C. The map of pHBAAV-CMV-circRNA-EF1-ZsGreen vector is shown in Table S4.

### Chondrocytes isolation and culture

Cartilage tissues were isolated from humans and rabbits, and treated with 0.2% type II collagenase (Sigma-Aldrich, USA) for 24 h at 37 °C. After filtering through a 0.075 mm cell strainer and centrifugation at 1500 rpm for 10 min, the precipitated cells were cultured in Dulbecco's Modified Eagle Medium (DMEM) supplemented with 10% FBS (Thermo Fisher Scientific, Waltham, MA, USA). The culture was maintained in an incubator set to 37°C with 5% CO_2_ and 100% humidity. The DMEM were changed and cells were washed with sterile phosphate buffered saline (PBS) every day during the first three days of culture. When the first-passage cells grown to occupy more than 80% of the bottom area of the culture dish, cells from one culture dish were digested with 1ml Trypsin and seeded into two culture dishes evenly as second-passage cells. The second-passage cells were used for transfection and other operations.

### Transfection

The siRNAs and the vectors that upregulate or downregulate relative miRNA expressions (miRNA mimic and miRNA inhibitor) were designed and constructed by RiboBio (Guangzhou, China). Lipofectamine RNAiMAX (ThermoFisher) was used for siRNA or miRNA mimic/inhibitor according to the manufacturer's instructions. Relative sequences are shown in Table S3.

### Virus infection

The overexpression plasmid of CircCDK14 (Sequences of CircCDK14 were inserted into XhoI restriction sites of pHBLV-CMV-Cicr-MCS-EF1-zsgreen-t2a-puro vector) and Smad2 (Sequences of Smad2 were inserted into EcoRI and BamHI restriction sites of pHB-CMV-MCS-EF1-zsgreen-t2a-puro vector) were designed and constructed by HanBio (Shanghai, China). In brief, restriction endonuclease XhoI, EcoRI and BamHI (Thermo Scientific) were used for vector cleavage to obtain a purified linearized vector. CircCDK14 and Smad2 fragments were amplified and ligated with the linearized vector, followed by transformation of DH5α competent cell. After cultivating with LB medium in culture plates for 12-16 h, the amplified sequence was detected by Sanger Sequencing to verify the consistency with CircCDK14 or Smad2. The plasmid was extracted and stored at -80 °C. Packaging plasmids and viral vectors were co-transfected into HEK-293T cells using Lipofectamine 3000 transfection reagent (ThermoFisher) according to the manufacturer's instructions. Forty-eight hours after transfection, the culture medium was centrifuged at 3000 rpm for 10 min and supplemented with 10 μg/mL polybrene (SolarBio). Finally, the culture medium mixture with polybrene was added to human chondrocytes (HC) and rabbit chondrocytes (RC), followed by selection with 2μg/mL puromycin in culture medium 36 h after infection. The maps of pHBLV-CMV-Cicr-MCS-EF1-zsgreen-t2a-puro vector and pHB-CMV-MCS-EF1-zsgreen-t2a-puro vector are shown in Table S4.

### Quantitative RT-PCR

Total RNA or miRNA were extracted from cartilage tissues or the treated cartilage cells using Ultrapure RNA Kit (CWBIO) or miRNA Purification kit (CWBIO) separately. Quantification of circRNA, mRNA and GAPDH gene were performed using UltraSYBR one step RT-qPCR Kit (CWBIO) according to the manufacturer's instructions. MiRNA qPCR Assay Kit (CWBIO) was used to detect the concentration of miRNA and U6. Each sample is repeated three times independently. The quantities of CircCDK14 and mRNA were normalized to GAPDH, and the expression levels of miRNA were normalized to U6. Relative primers are shown in Table S1.

### Western blot

Cartilage cells or tissues were lysed with radio immunoprecipitation assay buffer (RIPA, Beyotime, China) supplemented with 100 mM phenylmethanesulfonyl fluoride (PMSF) on ice. Bicinchoninic acid (BCA) analysis (Beyotime, China) was used to qualify the concentration of total proteins. Proteins were then separated by 8% SDS-PAGE and transferred to PVDF membranes (Bio-Rad), followed by blocking with 5% nonfat milk at room temperature for 1 h. After incubating with primary antibody at 4°C overnight, the membranes were washed by TBST and incubated with a secondary antibody at room temperature for 1 h. Protein bands were visualized using FDbio-Femto ECL (Fudebio, Hangzhou, China) and a chemiluminescence system (Bio-Rad, USA). Finally, the western blot density was quantified using Image J software (NIH). Three representative images from each group were converted to gray scale image, the data was expressed as intergral optical density (IOD) and normalized to negative control group.

### RNA FISH

Cy3-labeled CircCDK14 probes and fluorescein amiditelabeled miR-125a-5p probes were designedand synthesized by RiboBIO (Guangzhou, China). Cells were seeded into 6-well plates with cell climbing slice at a density of 2×10^5^ cells/well. After being fixed with 4% paraformaldehyde for 30 min and permeated with 0.5% tritonX-100 for 10 min, the probe signals were detected using a FISH kit (RiboBIO) according to the manufacturer's instruction. All operations were carried out in the dark. Prehybridization solution (1mL) was added to each well and blocked at 37 °C for 30 min. A volume of 500 μl hybridization solution containing Cy3-labeled CircCDK14 probes or fluorescein amiditelabeled miR-125a-5p probes were added to each well and incubated overnight at 37 °C to bind CircCDK14 and miR-125a-5p, followed by washing with hybrid washing solutions and PBS at 42 °C. The nuclei were stained with DAPI. For *in vivo* FISH, cartilage specimens were fixed in 4% paraformaldehyde for paraffin embedding and then sectioned at 5μm, tissue sections were deparaffinised, rehydrated, and permeabilized using a 0.8% pepsin treatment for 30 min at 37 °C before hybridization. All images were acquired using Nikon A1Si Laser Scanning Confocal Microscope (Nikon Instruments Inc, Japan). Semiquantitative analyses of the fluorescence intensity were performed using Image-Pro Plus 6.0 (NIH, Bethesda, MD, USA). Three representative images from each group were used to calculate the mean fluorescence intensity. The measurement parameters included sum of total area (Area), total cell number (CN), background average fluorescence intensity (BAFI) and integral fluorescence intensity (IFI). The image was converted to gray scale image and the values were quantified. The data was expressed as average IFI per cell ((IFI-Area*BAFI)/CN) and normalized to negative control group. The probe sequences are shown in Table S3.

### Bioinformatics analysis

The miRNA target of CircCDK14 was predicted by two bioinformatics databases Targetscan (http://www.targetscan.org/) and Rnahybrid (https://bibiserv.cebitec.uni-bielefeld.de/rnahybrid/, p-value < 0.05). The mRNA target of miR-125a-5p was by four bioinformatics databases Targetscan (http://www.targetscan.org/), Starbase (http://starbase.sysu.edu.cn/), miRTarbase (http://mirtarbase.mbc.nctu.edu.tw/) and miRwalk (http://mirwalk.umm.uni-heidelberg.de/, energy<-20, accessibility>0.01).

### RNA immunoprecipitation (RIP)

RIP experiments were performed using the Magna RIP RNA-Binding Protein Immunoprecipitation Kit (Millipore, Billerica, MA, USA). HEK-293T cells were transfected with the Ago2 plasmid or vector. Approximately 1 × 10^7^ cells were then subjected to an equal pellet volume and resuspended in 100 μl of RIP Lysis Buffer combined with protease inhibitors cocktail and RNase inhibitors. The cell lysates were incubated with antibody against Ago2 (Millipore) or IgG and rotated at 4 °C overnight. After treating with proteinase K buffer, the immunoprecipitated RNA were extracted by a RNeasy MinElute Cleanup Kit (Qiagen) and reverse transcribed (CWBIO). The expression levels of CircCDK14 were determined by qRT-PCR.

### Pull-down assay with biotinylated CircCDK14 probe

The biotinylated CircCDK14 probe was designed and synthesized by RiboBIO (Guangzhou, China). Approximately 1 × 10^7^ HC cells were harvested, lysed, and sonicated. CircCDK14 probe and Oligo probe were incubated into C-1 magnetic beads (Life Technologies) at 25 °C for 2 h to generate probe- coated beads. The cell lysates were incubated with these probe-coated beads at 4 °C overnight.

The RNA complexes bound to the beads were eluted and extracted with the RNeasy Mini Kit (QIAGEN) for RT-PCR or qRT-PCR after washing thrice with wash buffer. Relative probe sequence is shown in Table S3.

### Luciferase reporter assay

The luciferase reporter plasmid (Sequences of CircCDK14 or Smad2 and the mutant version were inserted into Xbal restriction sites of Firefly_Luciferase-Renilla_Luciferase vector) were constructed by Genechem (Shanghai, China). In brief, the vector Firefly_Luciferase-Renilla_Luciferase was select for use in this study, and restriction endonuclease XbaI (NEB) was used for vector cleavage to obtain a purified linearized vector. Target gene fragments were amplified and ligated with the linearized vector, followed by transformation of DH5α competent cell. After cultivating with LB medium in culture plates for 12-16 h, the amplified sequence was detected by Sanger Sequencing to verify the consistency with the target genes. The plasmid was extracted and stored at -80 °C. HEK-293T cells were seeded into 24-well plates and cultured to approximately 70% confluence, luciferase reporter plasmid and miR-125a-5p mimic/negative control mimic (RiboBio, Guangzhou, China) were co-transfected into HEK-293T using Lipofectamine 3000 transfection reagent (ThermoFisher) according to the manufacturer's instructions. Forty-eight hours after co-transfection, a dual luciferase reporter assay system (Promega, Madison, WI) was performed to measure the luciferase activity. And firefly luciferase activity was normalized to Renilla luciferase activity for calculation. The map of Firefly_Luciferase-Renilla_Luciferase vector is shown in Table S4.

### TUNEL staining assay

TUNEL staining was performed using the *In situ* Cell Death Detection kit, POD (Sigma-Aldrich) according to the manufacturer's protocol. In brief, cartilage specimens were fixed in 4% paraformaldehyde for paraffin embedding and sectioned at 5 μm. The sections were then deparaffinized with xylene and ethanol and the cells were rehydrated with proteinase K. After washing thrice with PBS, sections were incubated with TUNEL reaction mixture for 2 h at 37 °C in a moist chamber. The nuclei were stained with DAPI. All images were acquired using a fluorescence microscope (Eclipse E600; Nikon Corporation, Tokyo, Japan).

### EdU staining assay

Cells were transfected with siRNA or miRNA mimic/inhibitor for 48 h, then seeded into 96-well plates at a density of 1×10^4^ cells/well. After being incubated with 20μM EdU working solution for 12 h, EdU staining was performed using an EdU Imaging Test Kit (KeyGEN BioTECH). Briefly, cells were fixed with 4% paraformaldehyde for 30 min, neutralized with 2mg/mL glycine for 5 min, permeated with 0.5% tritonX-100 for 20 min and then incubated with EdU reaction mixture for 30 min in the dark. The nuclei were stained with DAPI. All images were acquired using a fluorescence microscope (Eclipse E600; Nikon Corporation, Tokyo, Japan).

### CCK8 cell proliferation assay

For the cell proliferation assay, cells were transfected with siRNA or miRNA mimic/inhibitor for 48 h, then seeded into 96-well plates at a density of 2×10^3^ cells/well and incubated for 0, 24, 48 or 72 h. At each time point, cell proliferation was detected using CCK8 (Sigma-Aldrich). Versamax microplate reader (Molecular Devices, CA, USA) was used to measure the absorbance of solution at 450 nm after 4 h of incubation.

### Cell apoptosis analysis by flow cytometry

For cell apoptosis analysis, cells were seeded into 6-well plates at a density of 2×10^5^ cells/well and received different treatments. An Annexin V-FITC/propidium iodide (PI) kit (BD Biosciences, SanDiego, CA, USA) was used according to the manufacturer's instructions. Cells were analyzed using a flow cytometer (BD FACSCANTO II, BD Biosciences, San Jose, CA, USA) and FlowJo software.

### Safranin O-fast green staining and OARSI scoring

Cartilage specimens were fixed in 4% paraformaldehyde for paraffin embedding and sectioned at 5 μm. Every tenth section was stained with 0.1% safranin O solution and 0.001% Fast Green solution (Sigma-Aldrich, St. Louis, MO, USA). The Osteoarthritis Research Society International (OARSI) score was based on safranin O-fast green staining of each specimen 29, the four different compartments of the joints, including medial femur, medial tibia, lateral femur and lateral tibia were assessed and the score of the most severe part was took as the score of the entire joint. Specific scoring data was shown in Table S2.

### Immunohistochemistry

Cartilage specimens were fixed in 4% paraformaldehyde for paraffin embedding and sectioned at 5μm. The sections were incubated with primary antibody at 4 °C overnight, washed thrice with PBST and incubated with a secondary antibody (Beyotime Institute of Biotechnology, Inc., Jiangsu, China) for 2 h at room temperature. The positively stained cells on the entire articular surface per specimen were counted, and the percentage of positive cells was calculated by Image-Pro Plus 6.0 (NIH, Bethesda, MD, USA). The number of cells positive for the marker was expressed relative to the total number of cells and the assessment was independently reviewed in parallel by two experienced pathologists.

### Immunofluorescence (IF)

Cells were fixed with 4% paraformaldehyde for 30 minutes, permeated with 0.5% tritonX-100 for 30 min and blocked with 5% bovine serum albumin (BSA) for 1 h. The cells were then incubated with primary antibodies (diluted 1:100 by BSA) at 4°C overnight, washed thrice with PBS and incubated with CL594- or CL488-conjugated secondary antibodies (Proteintech Group, Rosemount, IL, USA, diluted 1:200 by BSA) at room remperature for 1 h. Finally, after washing thrice with PBS, the nuclei were stained with DAPI. All operations, starting with the incubation of secondary antibodies, were performed in the dark. All images were acquired using a fluorescence microscope (Eclipse E600; Nikon Corporation, Tokyo, Japan). Semiquantitative analyses of the fluorescence intensity were performed using Image-Pro Plus 6.0 (NIH, Bethesda, MD, USA). Three representative images from each group were used to calculate the mean fluorescence intensity. The measurement parameters included sum of total area (Area), total cell number (CN), background average fluorescence intensity (BAFI) and integral fluorescence intensity (IFI). The image was converted to gray scale image and the values were quantified. The data was expressed as average IFI per cell ((IFI-Area*BAFI)/CN) and normalized to negative control group.

### Statistical analysis

Statistical analysis was performed using the SPSS version 18.0 software (IBM Corporation, USA). Data were analyzed with Student's t-test, Fisher's exact test, and one-way ANOVA. The results are presented as the mean ± SD. Group differences were considered statistically different for p<0.05 between groups.

## Results

### CircCDK14 expression is downregulated in weight-bearing area of joints and predominantly localized in the cytoplasm

**Figure 1:**
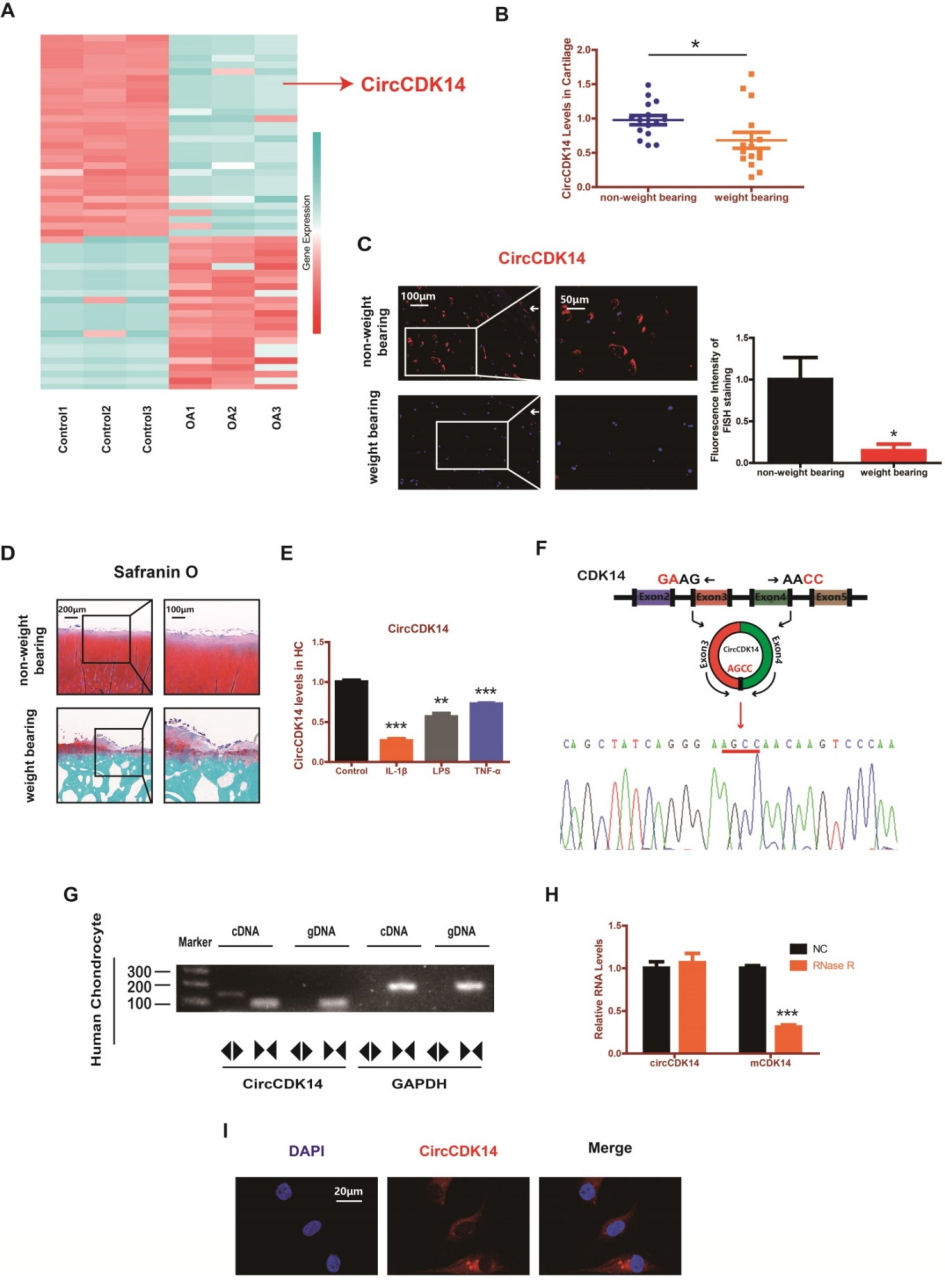
CircCDK14 expression is downregulated in weight-bearing area and predominantly localized in the cytoplasm. (**A**) The validation and expression of CircCDK14 in OA tissues. (**B**) The expression level of CircCDK14 in different stress areas of 15 human cartilage samples (n=15) *p<0.05. (**C**) Expression levels of CircCDK14 was detected by RNA FISH in weight bearing area and non-weight bearing area in human samples. Representative photomicrographs and fluorescence intensity of FISH are shown. (n=3) Scale bars, 100 µm and 50 µm. *p<0.05. Articular surface (while arrow) (**D**) Safranin-O/fast green staining of the cartilage from different stress areas of human sample. Scale bars, 200 µm and 100 µm. (**E**) Expression of CircCDK14 after treating with IL-1β (10ng/mL), TNF-α (10ng/mL) or LPS (500ng/mL) for 24 h. (n=3) **p<0.01, ***p<0.001. (**F**) Schematic illustration showing the CDK14 exon 3-4 circularization forming circCDK14 (black arrow). The presence of CircCDK14 was validated by qRT-PCR, followed by Sanger sequencing. Red arrow represents “head-to-tail” CircCDK14 splicing sites. (**G**) The presence of CircCDK14 was validated in HCs, CircCDK14 was amplified by divergent primers in cDNA but not gDNA. GAPDH was used as a negative control. (**H**) After treating or without treating with Rnase R, the expression of CircCDK14 and CDK14 mRNA in HCs were detected by qRT-PCR. (n=3) ***p<0.001. (**I**) RNA FISH showed that CircCDK14 was predominantly localized in the cytoplasm. CircCDK14 probes were labeled with Cy-3. The nuclei were stained with DAPI. Scale bar, 20 µm.

RNAseq analyses comparing circRNAs in three OA tissues with those in three control tissues were performed by previous research 18. We found that the expression level of CircCDK14 (also referred to as hsa_circ_0001722) was significantly downregulated in OA tissues (Figure 1A). To further verify this result, 15 human cartilage samples were collected wherein CircCDK14 was significantly downregulated in weight-bearing zone of joints compared with the non-weight bearing zone (Figure 1B-D). Considering that the degree of arthritis and inflammation in the weight-bearing area of joints was more severe, we hypothesized that the expression of CircCDK14 would decrease in an inflammatory environment. Therefore, human chondrocytes (HC) were abstracted and cultured from human cartilage samples and IL-1β, LPS or TNF-α were added to simulate the microenvironment of joint inflammation *in vivo*. Our results showed that CircCDK14 expression decreased substantially after being treated with IL-1β, LPS or TNF-α (Figure 1E), and the extent of the decrease was inversely proportional to the processing time and concentration of IL-1β (Figure S3A), which further confirmed our speculation that inflammation causes a decrease in the expression of CircCDK14. We also confirmed the head-to-tail splicing via Sanger sequencing (Figure 1F), as shown in Figure S2A-B, the whole sequence of CircCDK14 detected by Sanger sequencing was shown to be perfectly matched with the sequence in Circbase. However, head-to-tail splicing may be produced by trans-splicing or genomic rearrangement. To rule out these two possibilities, we further designed convergent primers to amplify CDK14 mRNA and divergent primers to amplify CircCDK14. CircCDK14 was shown to be only amplified by divergent primers and was detected in cDNA but not in genomic DNA (gDNA), whereas CDK14 mRNA could be amplified by convergent primers both in cDNA and gDNA (Figure 1G). Moreover, CDK14 mRNA was considerably decreased under the Rnase R treatment, but CircCDK14 was resistant to Rnase R treatment due to its covalently closed-loop structure (Figure 1H). RNA fluorescence *in situ* hybridization (FISH) revealed that CircCDK14 was predominantly localized in the cytoplasm (Figure 1I).

### Role of CircCDK14 on ECM metabolism, cell proliferation and cell apoptosis in chondrocytes

**Figure 2:**
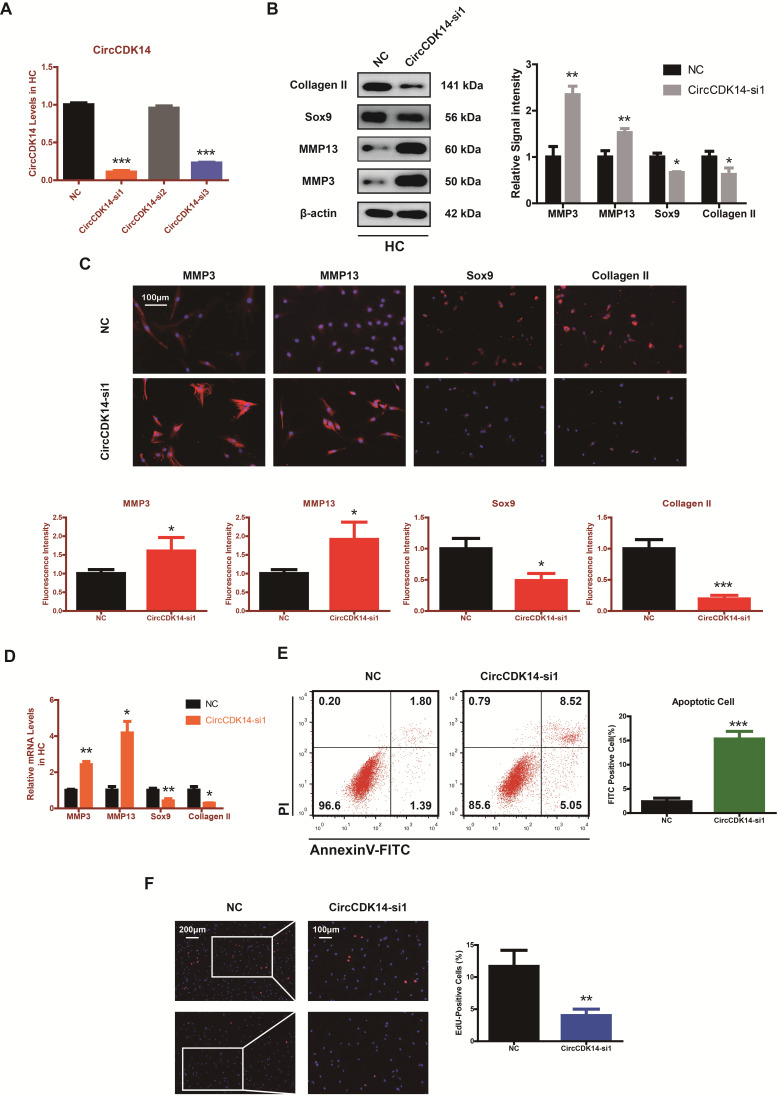
Knock-down of CircCDK14 on ECM metabolism, cell proliferation and cell apoptosis in chondrocytes. (**A**) The knock down efficiency of CircCDK14 in HCs was detected by qRT-PCR. (n=3) ***p<0.001. (**B**) Western blot analysis of MMP3, MMP13, Sox9 and Collagen II when CircCDK14 was downregulated in HCs. The optical density analysis was performed from the results of three independent experiments of western blot samples. *p<0.05, **p<0.01. (**C**) Representative photomicrographs and fluorescence intensity of IF of MMP3, MMP13, Sox9 and Collagen II when CircCDK14 was downregulated in HCs. (n=3, scale bar, 100 µm). *p<0.05, ***p<0.001. (**D**) The expression levels of MMP3, MMP13, Sox9 and Collagen II was detected by qRT-PCR when CircCDK14 was downregulated in HCs. (n=3) *p<0.05, **p<0.01. (**E**) HCs were transfected with si-CircCDK14, followed by Flow cytometry assay. The percentages of apoptotic cells are shown. (n=3) ***p<0.001. (**F**) HCs proliferation activity was detected by EdU staining when CircCDK14 was downregulated. Representative photomicrographs and quantitative data showing the percentage of EdU-positive cells are shown. (n=3, Scale bars, 200 µm and 100 µm). **p<0.01.

**Figure 3:**
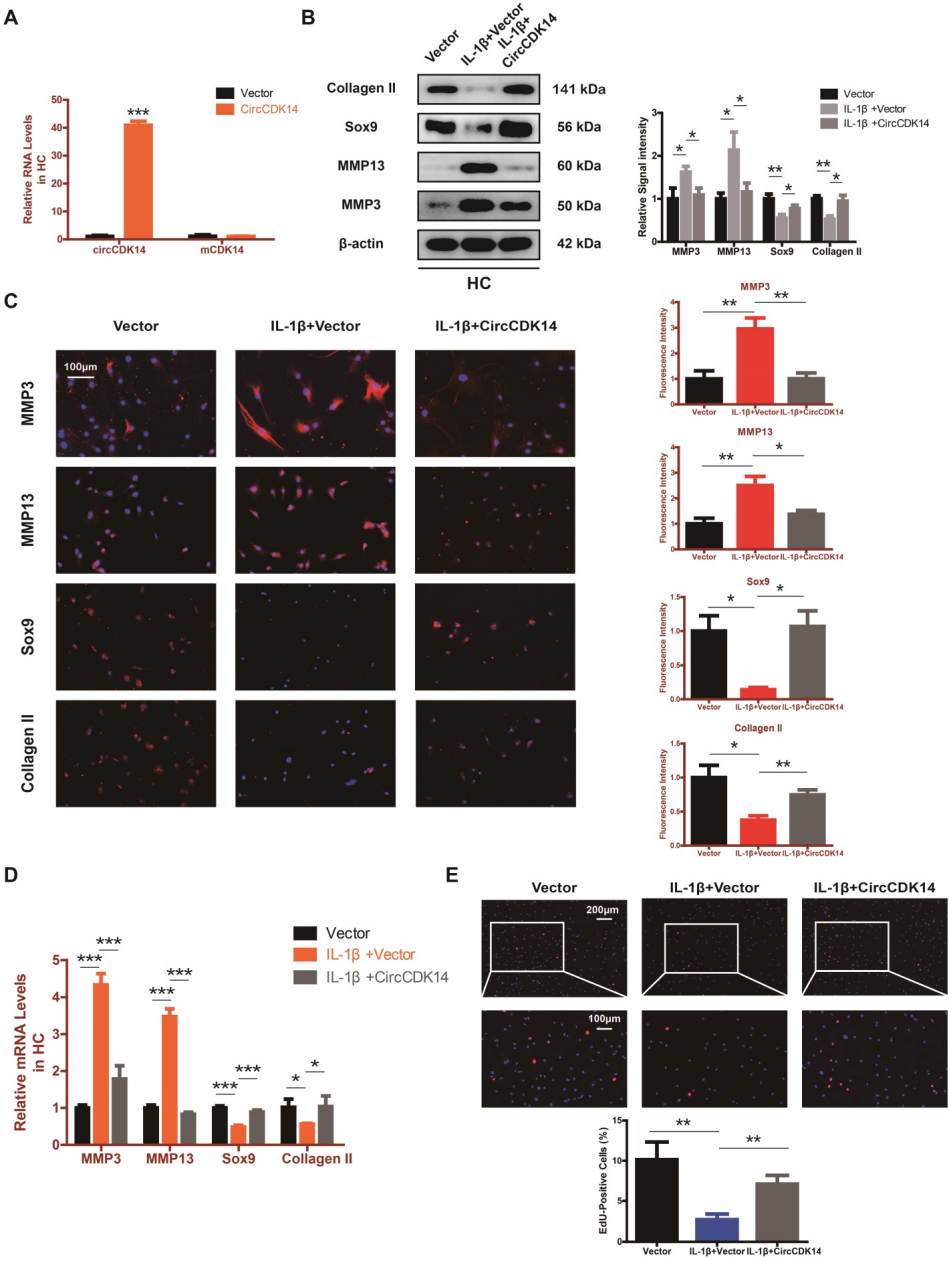
Overexpression of CircCDK14 on ECM metabolism, cell proliferation and cell apoptosis in IL-1βtreated chondrocytes. (**A**) The overexpression efficiency of CircCDK14 in HCs detected by qRT-PCR. (n=3) ***p<0.001. (**B**) Western blot analysis of MMP3, MMP13, Sox9 and Collagen II in HCs after treating with IL-1β (10 ng/ml) for 24 hours and the saving effects of overexpressed CircCDK14 on IL-1β. The optical density analysis was performed from the results of three independent experiments of western blot samples. *p<0.05, **p<0.01. (**C**) Representative photomicrographs and fluorescence intensity of IF of MMP3, MMP13, Sox9 and Collagen II in HCs after treating with IL-1β (10 ng/ml) for 24 hours and the saving effects of overexpressed CircCDK14 on IL-1β. (n=3 scale bar, 100μm) *p<0.05, **p<0.01. (**D**) The expression levels of MMP3, MMP13, Sox9 and Collagen II in HCs was detected by qRT-PCR after treating with IL-1β (10ng/ml) for 24 hours and the saving effects of overexpressed CircCDK14 on IL-1β. (n=3) *p<0.05, ***p<0.001. (**E**) HCs proliferation activity was detected by EdU staining in the above three groups. Representative photomicrographs and quantitative data showing the percentage of EdU-positive cells are shown. (n=3 Scale bars, 200 µm and 100 µm). **p<0.01.

To explore the role of CircCDK14 in chondrocytes, small-interfering RNA (siRNA) specific to CircCDK14 was transfected to knock down the expression level of CircCDK14. As shown in Figure 2A and Figure S3B, we verified the success of CircCDK14 knockdown and ensured that CDK14 mRNA was not affected. Western blot, qRT-PCR and immunofluorescence (IF) showed that the downregulation of CircCDK14 could promote the expression of MMP3 and MMP13, while decreasing that of Sox9 and Collagen II (Figure 2B-D, S7A, S8A). We then examined the effect on the proliferative capacity and found that CircCDK14 knockdown decreased cell proliferation of HCs (Figure 2F, S3C). Moreover, the results from the flow cytometry analysis of the effects of CircCDK14 on chondrocyte apoptosis showed that CircCDK14 silencing considerably enhanced the apoptosis rate (Figure 2E). To further explore the therapeutic effect of CircCDK14, the overexpression virus was constructed and transfected to upregulate CircCDK14 expression while CDK14 mRNA was not affected (Figure 2A, S4A). Weatern blot, qRT-PCR and IF showed that the treatment of IL-1β could promote the expression of MMP3 and MMP13 while decreasing that of Sox9 and Collagen II. Additionally, our results showed that the overexpression of CircCDK14 was able to antagonize this effect of IL-1β (Figure 3B-D, s4b, s4c, s7b, s8b). As shown in Figure 3E and Figure S3D, the reduced proliferative capacity after IL-1β treatment was markedly increased in response to CircCDK14 overexpression. QRT-PCR showed that the overexpression of CircCDK14 inhibited IL-1β-induced inflammatory response in HC (S3K). Taken together, these results indicated that CircCDK14 could protect against OA by regulating cell proliferation, cell apoptosis and ECM metabolism in chondrocytes.

### CircCDK14 efficiently sponges miR-125a-5p in HC

**Figure 4:**
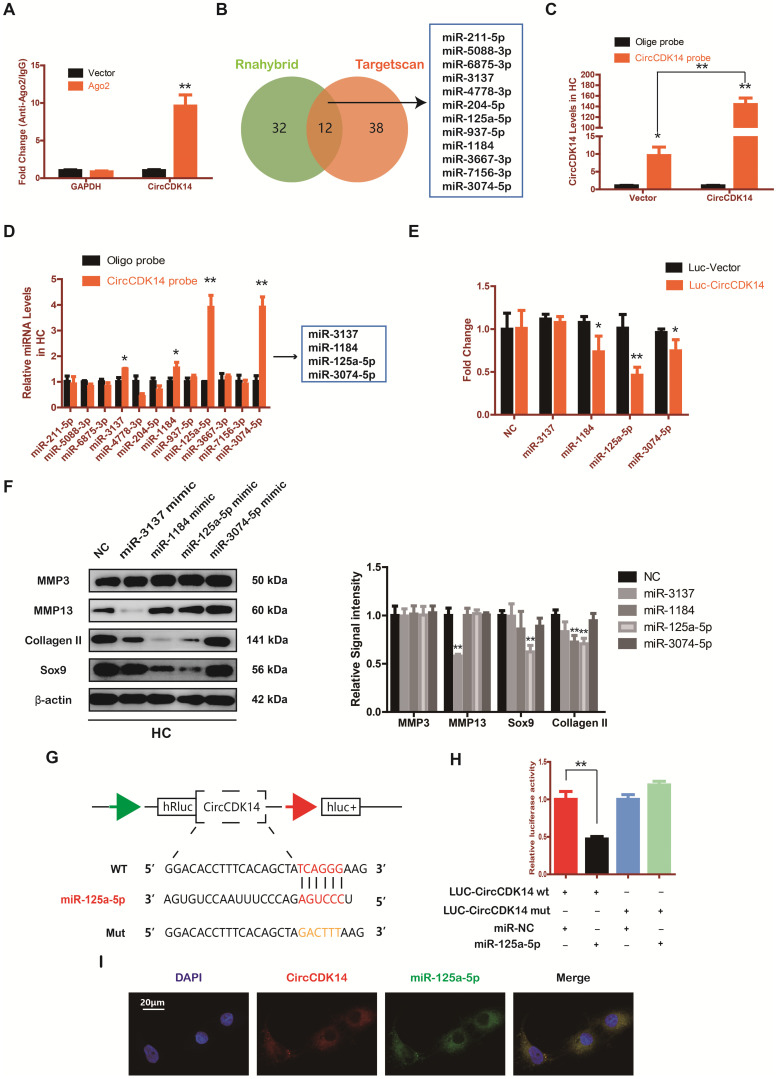
CircCDK14 efficiently sponges miR-125a-5p in HC. (**A**) RNA immunoprecipitation (RIP) assay for circTADA2A levels in HEK-293 cells transfected with Ago2. (n=3) **p<0.01. (**B**) Schematic illustration to show the overlapping of the target miRNAs of CircCDK14 predicted by Rnahybrid and Targetscan, and 12 different miRNAs were included in our consideration. (**C**) Lysates prepared from HCs stably transfected with CircCDK14 or vector were subjected to RNA pull-down assays and tested by qRT-PCR. Relative levels of CircCDK14 pulled down by the CircCDK14 probe were normalized to the level of CircCDK14 pulled down by an oligo probe. (n=3) *p<0.05, **p<0.01. (**D**) The relative level of 12 miRNA candidates in the HC lysates was detected by qRT-PCR. (n=3) *p<0.05, **p<0.01. (**E**) Luciferase activity of the Luc-vector or Luc-CircCDK14 in HEK-293 T cells co-transfected with indicated 4 miRNA mimics. (n=3) *p<0.05, **p<0.01. (**F**) Western blot analysis of MMP3, MMP13, Sox9 and Collagen II when the above 4 candidate miRNAs were overexpressed respectively. The optical density analysis was performed from the results of three independent experiments of western blot samples. **p<0.01. (**G**) Schematic illustration demonstrates complementary to the miR-125a-5p seed sequence with CircCDK14. Yellow letters indicate mutated nucleotides. (**H**) MiR-125a-5p mimic or NC were co-transfected with a luciferase reporter construct containing CircCDK14 WT or CircCDK14 MUT into HEK-293T. The luciferase reporter activities are shown. (n=3) *p<0.05. (**I**) RNA FISH showed that CircCDK14 was co-localized with miR-125a-5p in HCs. CircCDK14 probes were labeled with Cy-3, miR-125a-5p were labeled with fam. The nuclei were stained with DAPI. Scale bar, 20 µm.

Accumulating evidence indicate that miRNA sponge is one of the major roles played by cytoplasm-located circRNAs 30-32. Given that CircCDK14 was mainly localized in the cytoplasm, we speculated that CircCDK14 acted by sponging a certain miRNA. To confirm our conjecture, HEK-293T cells were transfected with the Ago2 plasmid, or vector, and RNA immunoprecipitation was performed using antibody targeting Ago2. We demonstrated that endogenous CircCDK14 pulled-down from Ago2 antibodies was predominantly enriched in the Ago2 overexpression group compared with the control group using qRT-PCR (Figure 4A). Then two databases (targetscan and rnahybrid) were used to predict the potential target miRNAs and 12 miRNAs were selected by overlapping the prediction results (Figure 4B). Moreover, we designed a biotin-labeled CircCDK14 probe and verified the pull-down efficiency (Figure 4C). To investigate whether the 12 miRNAs were bound to CircCDK14 directly, expressions of the miRNAs were detected with qRT-PCR following the pull-down assay. As shown in Figure 4D, of the 12 candidate miRNAs, four of them (miR-3137, miR-1184, miR-125a-5p and miR-3074-5p) were pulled-down. We then constructed a luciferase reporter plasmid containing the full-length of CircCDK14. A luciferase assay that was performed to detect the binding of these four miRNAs showed that only miR-125a-5p had strongly reduced luciferase activity more than 50%, compared with the control (Figure 4E). We also examine the function of these miRNAs towards catabolism and anabolism. As shown in Figure 4F and Figure S8C, when miR-125a-5p was overexpressed, the expression of Collagen II and Sox9 was downregulated, while there were no changes in the expression of MMP3 and MMP13. Therefore, miR-125a-5p was selected for further analysis. We mutated the predicted miR-125a-5p-binding site on CircCDK14 and constructed another luciferase reporter plasmid containing CircCDK14 mutant vectors, co-transfected with miR-125a-5p mimic into HEK-293T cells. The luciferase reporter assay confirmed the binding of CircCDK14 to miR-125a-5p (Figure 4G-H). Furthermore, FISH indicated that miR-125a-5p was also predominantly localized in cytoplasm and roughly overlapped in spatial distribution with CircCDK14 (Figure 4I). These results confirmed the role of CircCDK14 as a sponge of miR-125a-5p.

### MiR-125a-5p is upregulated in weight-bearing area of joints and contrary to the role of CircCDK14

**Figure 5:**
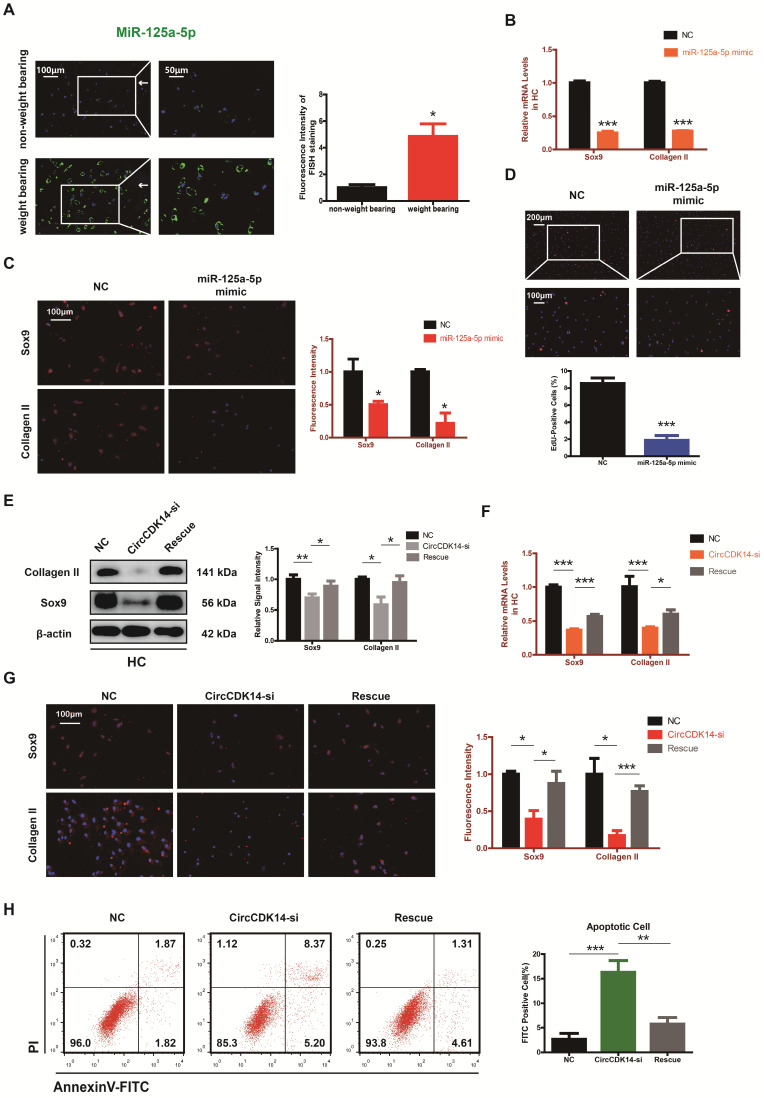
MiR-125a-5p is upregulated in weight-bearing area of joints and contrary to the role of CircCDK14. (**A**) MiR-125a-5p expression was detected by RNA FISH in different stress areas of human sample. Representative photomicrographs and fluorescence intensity of FISH are shown. (n=3) *p<0.05. Scale bars, 100 µm and 50 µm. Articular surface (while arrow) (**B**) The expression levels of Sox9 and Collagen II were detected by qRT-PCR when miR-125a-5p was upregulated in HCs. (n=3) ***p<0.001. (**C**) Representative photomicrographs and fluorescence intensity of IF of Sox9 and Collagen II in HC when miR-125a-5p was upregulated in HCs. (n=3 Scale bar, 100 µm) *p<0.05. (**D**) HCs proliferation activity was detected by EdU staining when miR-125a-5p was upregulated. Representative photomicrographs and quantitative data showing the percentage of EdU-positive cells are shown. (n=3 Scale bars, 200 µm and 100 µm). ***p<0.001. (**E-G**) WB, qRT-PCR and IF showed that the downregulation of miR-125a-5p antagonized the effect of CircCDK14-si on Sox9 and Collagen II in HCs (qRT-PCR n=3 *p<0.05, ***p<0.001). The optical density analysis was performed from the results of three independent experiments of western blot samples. (n=3) *p<0.05, **p<0.01. Representative photomicrographs and fluorescence intensity of IF are shown. (n=3 Scale bar, 100 µm) *p<0.05, ***p<0.001. (Rescue, CircCDK14-si+miR-125a-5p inhibitor) (**H**) Flow cytometry experiment indicated that the overexpression of miR-125a-5p could decrease HCs apoptosis rate induced by knockdown of CircCDK14. (n=3) **p<0.01, ***p<0.001.

The expression level of miR-125a-5p was detected using RNA FISH in human cartilage samples. Our study revealed that miR-125a-5p had a significantly higher expression in the joint weight bearing area (Figure 5A). Western blot, qRT-PCR and IF showed that miR-125a-5p overexpression could downregulate the expression of Collagen II and Sox9 (Figure 5B-C, S4D-E, S7C, S8D). Cell proliferation assay indicated that the expression level of miR-125a-5p was negatively associated with cell proliferative capacity (Figure 5D, S3E).

Moreover, when siRNA for CircCDK14 was co-transfected with miR-125a-5p inhibitor, the effects of CircCDK14 knockdown on ECM anabolism, cell proliferation and cell apoptosis in HC were reversed (Figure 5E-H, S3F, S7D, S8E). These results suggested that miR-125a-5p is an important downstream target for CircCDK14 but also plays an antagonizing role against CircCDK14 in the chondrocytes.

### Smad2 mediates the CircCDK14/miR-125a-5p axis in HC

**Figure 6:**
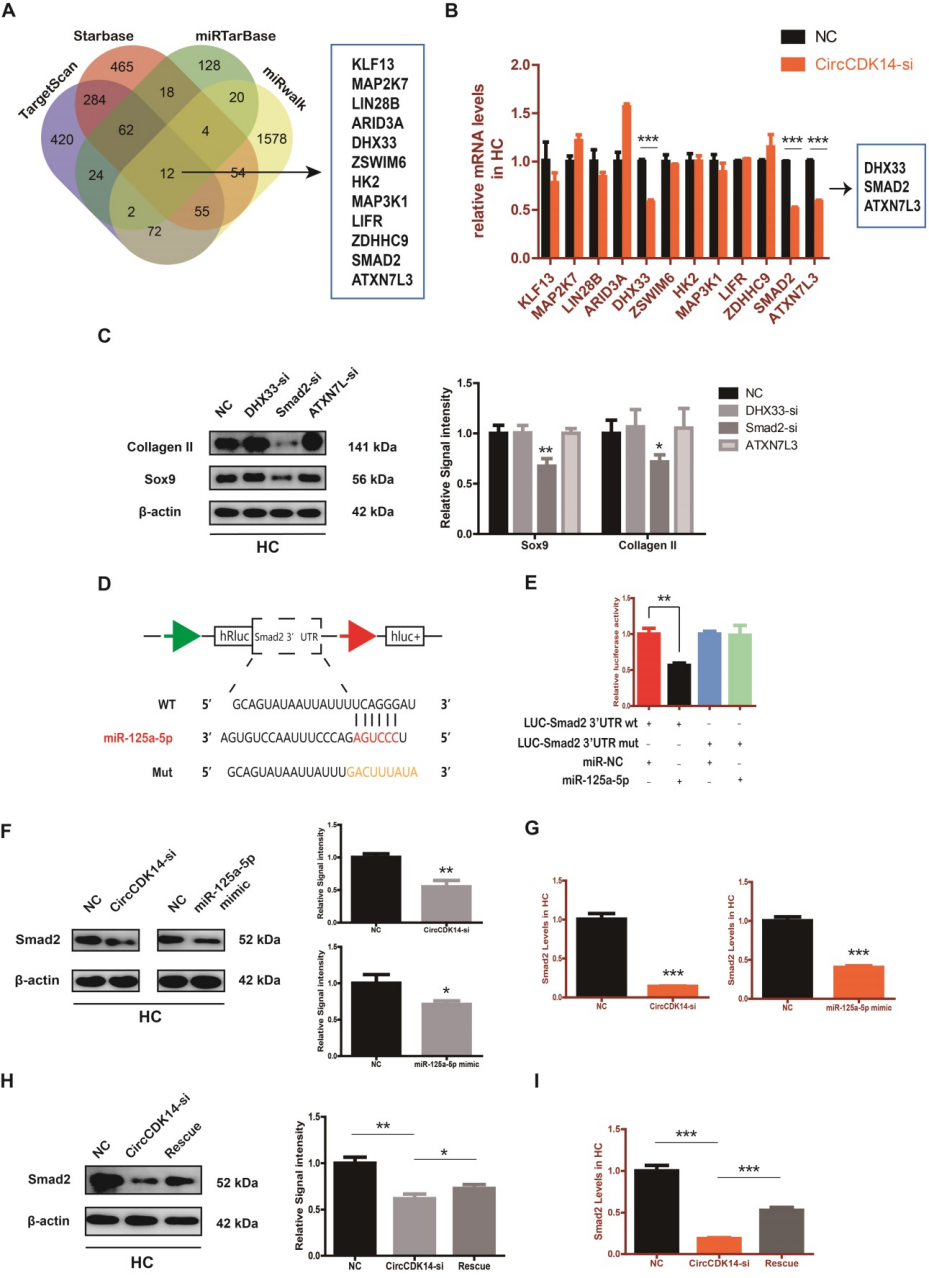
Smad2 serves as a direct target of miR-125a-5p. (**A**) Schematic illustration to show the overlapping of the target mRNAs of miR-125a-5p predicted by Targetscan, Starbase, miRTarBase and miRwalk. Twelve different mRNAs were considered. (**B**) Expression of 12 miRNAs were detected by qRT-PCR when CircCDK14 was downregulated in HCs. (n=3) ***p<0.001. Three mRNAs were downregulated. (**C**) Western blot analysis of Sox9 and Collagen II when the above three candidate mRNAs were downregulated respectively in HCs. The optical density analysis was performed from the results of three independent experiments of western blot samples (n=3) *p<0.05, **p<0.01. (**D**) Schematic illustration demonstrates complementary to the miR-125a-5p seed sequence with Smad2. Yellow letters indicate mutated nucleotides. (**E**) MiR-125a-5p mimic or NC were co-transfected with a luciferase reporter construct containing Smad2 WT or Smad2 MUT into HEK-293T, and the luciferase reporter activities of Smad2-mut was obviously higher than wt of Smad2. (n=3) **p<0.01. (**F-G**) Western blot and qRT-PCR analysis of Smad2 in HCs when treated with CircCDK14-si or miR-125a-5p mimic. (qRT-PCR n=3 ***p<0.001.) The optical density analysis was performed from the results of three independent experiments of western blot samples. (n=3) *p<0.05, **p<0.01. (**H-I**) WB and qRT-PCR showed that the downregulation of miR-125a-5p antagonized the effect of CircCDK14-si on Smad2 in HCs. (qRT-PCR n=3 ***p<0.001) The optical density analysis was performed from the results of three independent experiments of western blot samples. (n=3) *p<0.05, **p<0.01.

**Figure 7:**
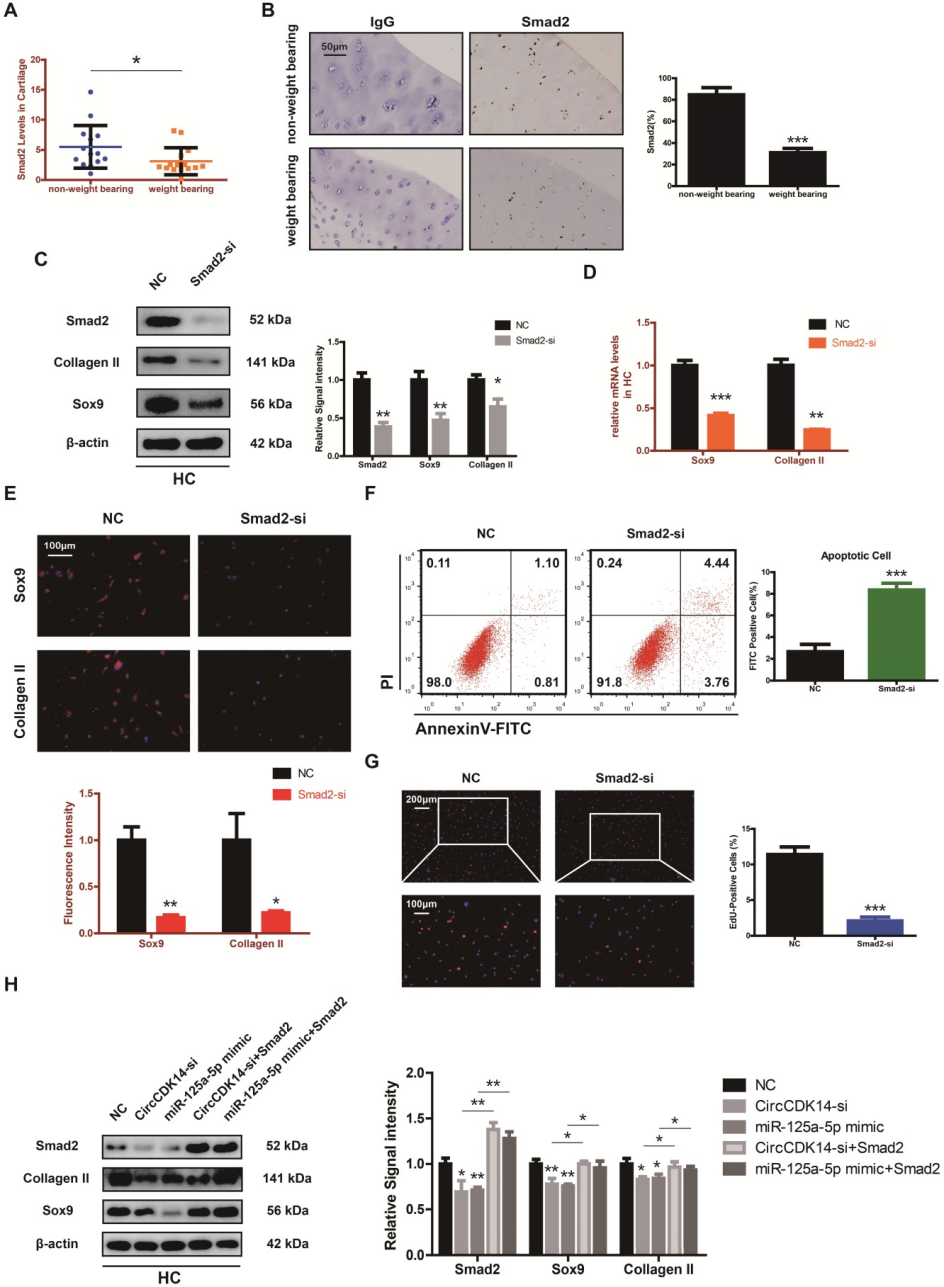
Smad2 mediates the CircCDK14/miR-125a-5p axis in HC. (**A**) The expression of Smad2 in different stress areas of 15 human cartilage samples (n=15) *p<0.05. (**B**) Expression of Smad2 were observed by immunohistochemistry staining in weight bearing areas compared with non-weight bearing areas of human samples. ***p<0.001. Scale bar, 50 µm. (**C**) Western blot analysis of Smad2, Sox9 and Collagen II when Smad2 was downregulated in HCs. The optical density analysis was performed from the results of three independent experiments of western blot samples (n=3) *p<0.05, **p<0.01. (**D-E**) The expression levels of Sox9 and Collagen II were detected by qRT-PCR and IF when treated with Smad2-si in HCs. (qRT-PCR n=3 **p<0.01, ***p<0.001). Representative photomicrographs and fluorescence intensity of IF are shown. (n=3 Scale bar, 100 µm) *p<0.05, **p<0.01. (**F**) Flow cytometry experiment indicated that siRNA-mediated Smad2 knockdown increased HCs apoptosis rate. (n=3) ***p<0.001. (**G**) HCs proliferation activity was detected by EdU staining when Smad2 was downregulated. Representative photomicrographs and quantitative data showing the percentage of EdU-positive cells are shown. (n=3 Scale bars, 200 µm and 100 µm). ***p<0.001. (**H**) Western blot analysis showed that overexpression of Smad2 could antagonize the effects of CircCDK14-si and miR-125a-5p mimic on Smad2, Sox9 and Collagen II in HCs. The optical density analysis was performed from the results of three independent experiments of western blot samples. (n=3) *p<0.05, **p<0.01.

To explore the downstream targets of CircCDK14/miR-125a-5p axis, we first selected 12 possible mRNAs by overlapping the predicted results of Targetscan, Starbase, miRTarBase and MiRwalk (Figure 6A). QRT-PCR results showed that three of them (DHX33, Smad2 and ATXN7L3) were down-regulated when CircCDK14 was downregulated (Figure 6B). Among the three candidates, only Smad2 had an effect on Sox9 and Collagen II (Figure 6C, S8H). Moreover, we found that Smad2 3' UTR contained sequences that are complementary to the miR-125a-5p seed sequence (Figure S2D). Next, luciferase reporter plasmids containing these complementary sequences, or mutant vectors of these sequences, were constructed and co-transfected into HEK293T cells with miR-125a-5p mimics. The results confirmed the binding of Smad2 to miR-125a-5p (Figure 6D-E). Western blot and qRT-PCR results showed that both knock-down of CircCDK14 and overexpression of miR-125a-5p decreased the expression of Smad2, while inhibition of miR-125a-5p could antagonize the effect of CircCDK14 knockdown (Figure 6F-I, S4G-H, S8F-G). Therefore, Smad2 was confirmed as an important downstream target for further research. As shown in Figure 7A-B, the expression level of Smad2 was obviously lower in joint load bearing zone in 15 human cartilage samples. Furthermore, to explore the function of Smad2 in OA development, siRNA specifically targeted at Smad2 was transfected to downregulate Smad2 expression. Our results confirmed the success of Smad2 knockdown both at the mRNA and protein levels (Figure 7C, S3G, S5A, S8I). Western blot, qRT-PCR and IF showed that Smad2 silencing remarkably downregulated the expression of Sox9 and Collagen II in chondrocytes (Figure 7C-E, S5B-C, S7E, S8I). Furthermore, knockdown of Smad2 decreased cell proliferative capacity in chondrocytes (Figure 7G, S3H) and increased chondrocytes apoptosis rate (Figure 7F). Moreover, the overexpression virus was constructed and transfected to upregulate Smad2 expression (Figure S3i). Western blot and qRT-PCR analysis showed that the overexpressed smad2 could antagonize the effects of CircCDK14 knockdown or miR-125a-5p mimic on Sox9 and Collagen II (Figure 7H, S3J, S5E-F, S8J). Taken together, our results revealed that Smad2 was the main mediator of the CircCDK14/miR-125a-5p axis, which exhibited a similar function as does CircCDK14 in chondrocytes.

### Injection of CircCDK14 alleviates OA *in vivo*

**Figure 8:**
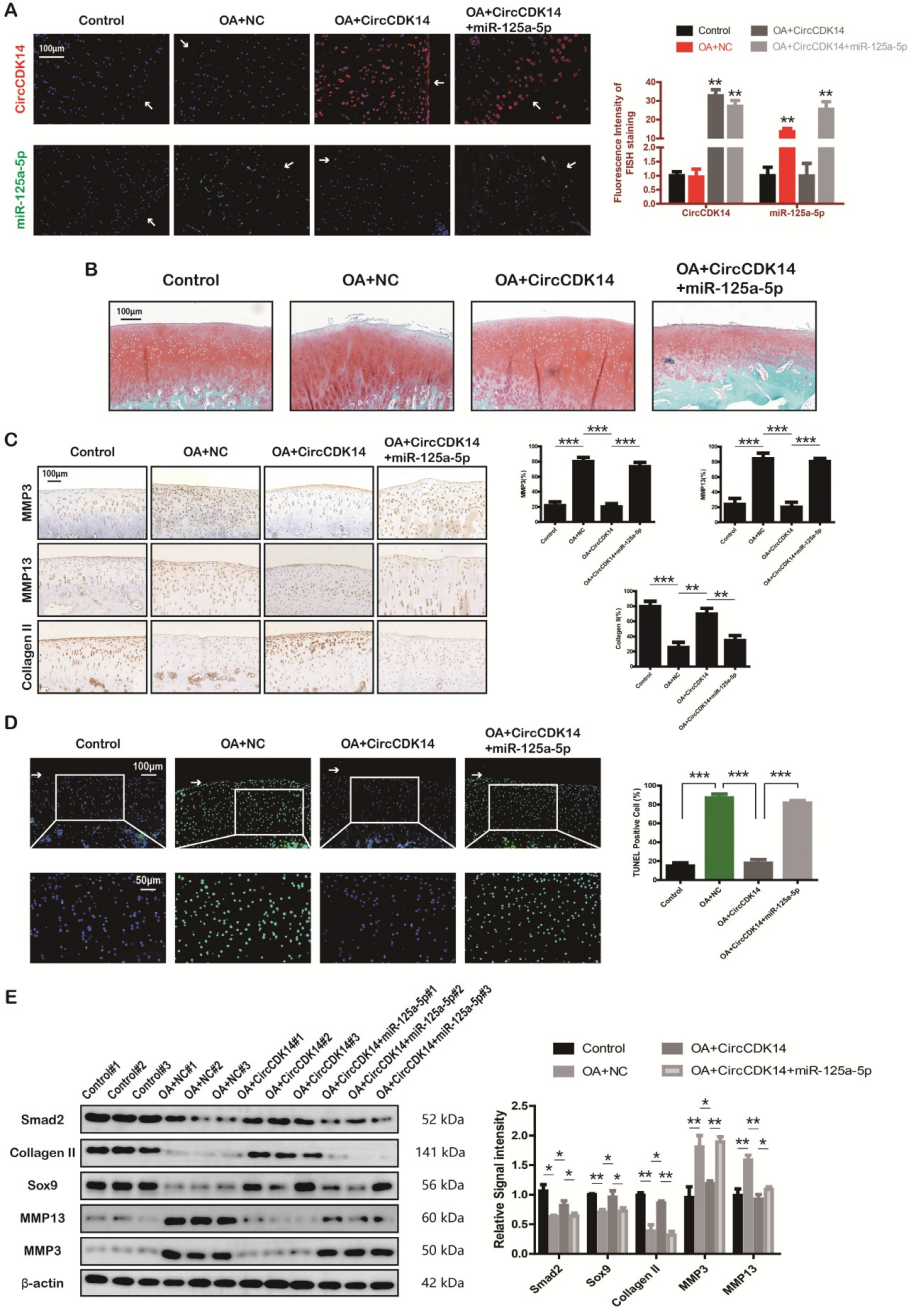
Injection of CircCDK14 alleviates OA *in vivo*. (**A**) Expression of CircCDK14 and miR-125a-5p in cartilage tissue from different groups (FISH). Representative photomicrographs and fluorescence intensity of FISH are shown. (n=6) Scale bar, 100 µm. **p<0.01. Articular surface (while arrow) (**B**) Safranin-O/fast green staining of cartilage from different groups. Scale bar, 100 µm. (**C**) Histological analysis of rabbit cartilage tissue in each group. MMP3, MMP13 and Collagen II expression was examined by immunohistochemistry**.** Scale bar, 100 µm. **p<0.01, ***p<0.001. (**D**) TUNEL staining of rabbit cartilage tissues in each group. Representative photomicrographs and quantitative data showing the percentage of TUNEL-positive cells are shown. (n=3) Scale bar, 100 µm and 50 µm. **p<0.01. Articular surface (while arrow) (**E**) Western blot analysis of Smad2, MMP3, MMP13, Sox9 and Collagen II in each group. Three cartilage samples were selected from each group for western blot analysis. **p<0.05, **p<0.01.

To further investigate the effects of CircCDK14 *in vivo*, a rabbit model was introduced in this study as described in the Methods section (S2E, S6A). FISH and qRT-PCR were performed to evaluate the overexpression levels of CircCDK14, miR-125a-5p and Smad2 (Figure 8A, S6B). Safranin O and fast green staining showed the substantially thickened cartilage layer on the articular surface after the injection of WT AAV CircCDK14; however, the overexpression of miR-125a-5p weakened this effect (Figure 8B). Quantitative analysis with OARSI scoring showed that WT AAV CircCDK14 significantly lowered OARSI scores, but the addition of miR-125a-5p makes this scores to increase again (Figure S6C). Moreover, as indicated by immunohistochemistry (IHC) and Western blot analysis, the injection of WT AAV CircCDK14 decreased the expression of MMP3, MMP13 and increased Smad2, Sox9, and Collagen II levels in chondrocytes, while the overexpression of miR-125a-5p reduced the expression of Sox9 and Collagen II while increasing that of MMP3 and MMP13 (Figure 8C, 6E). Furthermore, the cell apoptosis (TUNEL staining assay) showed consistent results as *in vitro* experiments (Figure 8D). Together, our results indicated that CircCDK14 could alleviate OA *in vivo* by maintaining anabolism and inhibiting catabolism in the ECM of cartilage, and these effects were achieved by combining with miR-125a-5p.

## Discussion

Current treatment strategies for OA, such as joint replacement surgery, drug therapy, or other strategies mainly aim to relieve pain and improve function 33. For patients at the end-stage, joint replacement surgery has been the final and standard approach; however, this approach cannot radically cure OA. 1, 34. Quite notably, these therapies all focus on relieving symptoms, rather than modifying the progression of OA, and none of the current treatments are able to cure OA completely. Therefore, novel molecular targets for OA treatment are urgently needed. Identifying these targets may benefit from a deeper understanding of the mechanism underlying the development and progression of OA.

CircRNAs are a new class of endogenous noncoding RNAs enriched in higher eukaryotes. Its role in the development of various human diseases has been increasingly recognized 35, 36. Previously, most studies have focused on neoplastic diseases 37-41. However, more recently, increasing evidence indicates that circRNAs could play a role as biomarkers and therapeutic targets for degenerative diseases such as intervertebral disc degeneration (IVDD) 42, 43. Nevertheless, only few studies have expounded the role of circRNAs on OA, the joint degenerative disease which continues to have a wide impact worldwide. In our present study, we first identified a key circRNA (CircCDK14) in OA, which was downregulated in joint-wearing position of patients and was closely related to the development and progression of OA. Our results showed that CircCDK14 could protect against OA by targeting at miR-125a-5p and Smad2.

CircCDK14 is derived from exons 3 and 4 of CDK14 gene. As one of the gene regulators that were closely associated with cell proliferation and cell apoptosis, CDK14 has been shown to be related to tumor invasiveness and prognosis by a large number of studies 44-47. However, the function of CircCDK14, whether in physiological or pathological states, remains unknown. We found that CircCDK14 was downregulated in joint wearing zone, and treatment of IL-1β and other pro-inflammatory factors could decrease its expression in human chondrocytes *in vitro*. Moreover, by influencing ECM metabolism, cell proliferation and cell apoptosis in chondrocytes, the downregulation of CircCDK14 could also aggravate OA in turn, which has been shown to form a nausea cycle in the joint. To break this vicious cycle, we upregulated CircCDK14 expression both *in vitro* and *in vivo*, the results confirmed that the overexpression of CircCDK14 could protect against OA. Our rationale to use the rabbit models *in vivo* experiments was based on its similarity to human beings in the development of OA, wherein Smad2-binding sites of miR-125a-5p are highly conserved. Through injecting c virus containing human CircCDK14 into the joint cavity of rabbits, which could bind to miR-125a-5p in rabbits and affect the expression of Smad2, thereby protected against OA in rabbits. Furthermore, we also verified the function of miR-125a-5p and Smad2 in rabbit chondrocytes (RC) *in vitro*. Our study, by revealing the protective role and underlying mechanism of CircCDK14, proposed a novel and effective molecular target in the treatment of OA.

As an essential member of ceRNA networks, circRNAs was found to act as “miRNA sponge” 17, 30, 48. Previous studies have found that miR-125a-5p was associated with multiple diseases, including neoplastic diseases 49, 50, metabolic diseases 51 and cerebrovascular diseases^52^. Our studies further revealed that miR-125a-5p was upregulated in the joint weight bearing area and had an antagonizing effect against CircCDK14. Quantitative RT-PCR, luciferase assay and FISH were performed to evaluate the interaction of CircCDK14 and its downstream target (miR-125a-5p), as well as mRNA target (Smad2), a key regulator in TGF-β signaling pathway.

Canonical TGF-β signaling promotes the phosphorylation of Smad2 and Smad3. Phosphorylated Smad2 and Smad3 interact with Smad4 in the cytoplasm, where they translocate to the nucleus together to induce gene transcription 25, 53. Studies have demonstrated that TGF-β/Smad2 signals was closely related with cell proliferation, apoptosis and inflammation 54-56. And its role in cartilage was also widely studied 57-59. For example, Wang et al. 60 showed Smad2 could regulate chondrocytes proliferation and differentiation in the growth plate. Additionally, Zhang at al. 61 reported that Smad2 could induce Sox9 and Collagen II expression. Our present research revealed that Smad2 was downregulated in the joint wearing zone, and was regulated by both CircCDK14 and miR-125a-5p. Moreover, as an important downstream target of CircCDK14/miR-125a-5p axis, Smad2 overexpression was verified to play a protective role in OA.

However, both miR-125a-5p and Smad2 could only partially explain the effect of CircCDK14. CircCDK14/miR-125a-5p/Smad2 axis was able to protect against OA by regulating ECM anabolism, chondrocytes apoptosis and proliferation, but was not able to down-regulate the expression of MMP3 or MMP13. Therefore, it is rational to infer that other genes or regulation pathways may exist for CircCDK14 to play a role in inhibiting catabolism in OA development.

In conclusion, we unveiled a new signaling pathway, CircCDK14/miR-125a-5p/Smad2, that could protect against OA by maintaining the extracellular matrix of chondrocytes and regulating chondrocyte apoptosis and proliferation. Consequently, overexpression of CircCDK14 and Smad2 or inhibitor of miR-125a-5p may be a promising therapeutic approach to OA.

## Supplementary Material

Supplementary figures and tables.

## Abbreviations

OA: Osteoarthritis
ECM: extracellular matrix
HC: human chondrocyte
RC: rabbit chondrocyte
CircRNA: Circular ribonucleic acid
MiRNA: Micro ribonucleic acid
ceRNA: competing endogenous RNA
cDNA: complementary DNA
gDNA: genomic DNA
IF: immunofluorescence
FISH: fluorescence *in situ* hybridization
RIP: RNA immunoprecipitation
IHC: immunohistochemistry
ACLT: anterior cruciate ligament transection
IVDD: intervertebral disc degeneration
CDK: Cyclin-dependent kinase
OARSI: Osteoarthritis Research Society International
