# Supplementary material for: CircCDK14 protects against Osteoarthritis by sponging miR-125a-5p and promoting the expression of Smad2
Source: Theranostics. 2020 Jul 11;10(20):9113–31. doi: 10.7150/thno.45993 (PMC7415803; doi:10.7150/thno.45993)
Supplement: Supplementary file 1 — Supplementary figures and tables. [file thnov10p9113s1.pdf]

A

General Conditions of Patients	
Item	Non-weight bearing/weight bearing
Number of cases	15
Age	65.13± 9.32
Gender(M/F)	6/9
Outerbridge Grade	
Stage I	2
Stage II	4
Stage III	4
Stage IV	5

B

Outerbridge Grade	
Stage I	Localised softening of the cartilage with minimal or no break in the surface
Stage II	Fissuring within the softened area often longitudinal 'shark gill' type area < 1.25cm
Stage III	Breakdown of the surface and fibrillation, extending down to subchondral bone 'Crab meat' type Area > 1.25 cm
Stage IV	Early osteoarthritis; erosive changes and exposure of subchondral bone, usually involves the opposite articular surface (more often the lateral than the medial femoral surface).

C

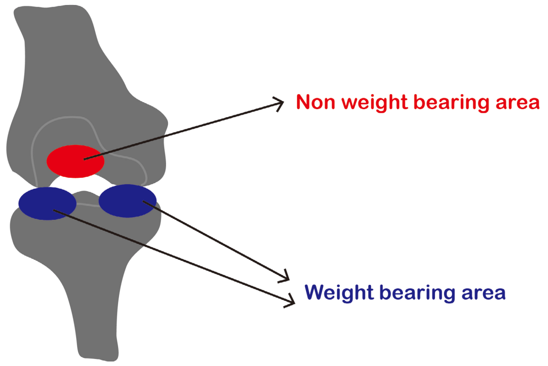

Figure S1 **(A)** General conditions of patients. **(B)** Standard of Outerbridge Grade. **(C)** Schematic diagram of the selection criteria for the knee weight-bearing area and non-weight-bearing area

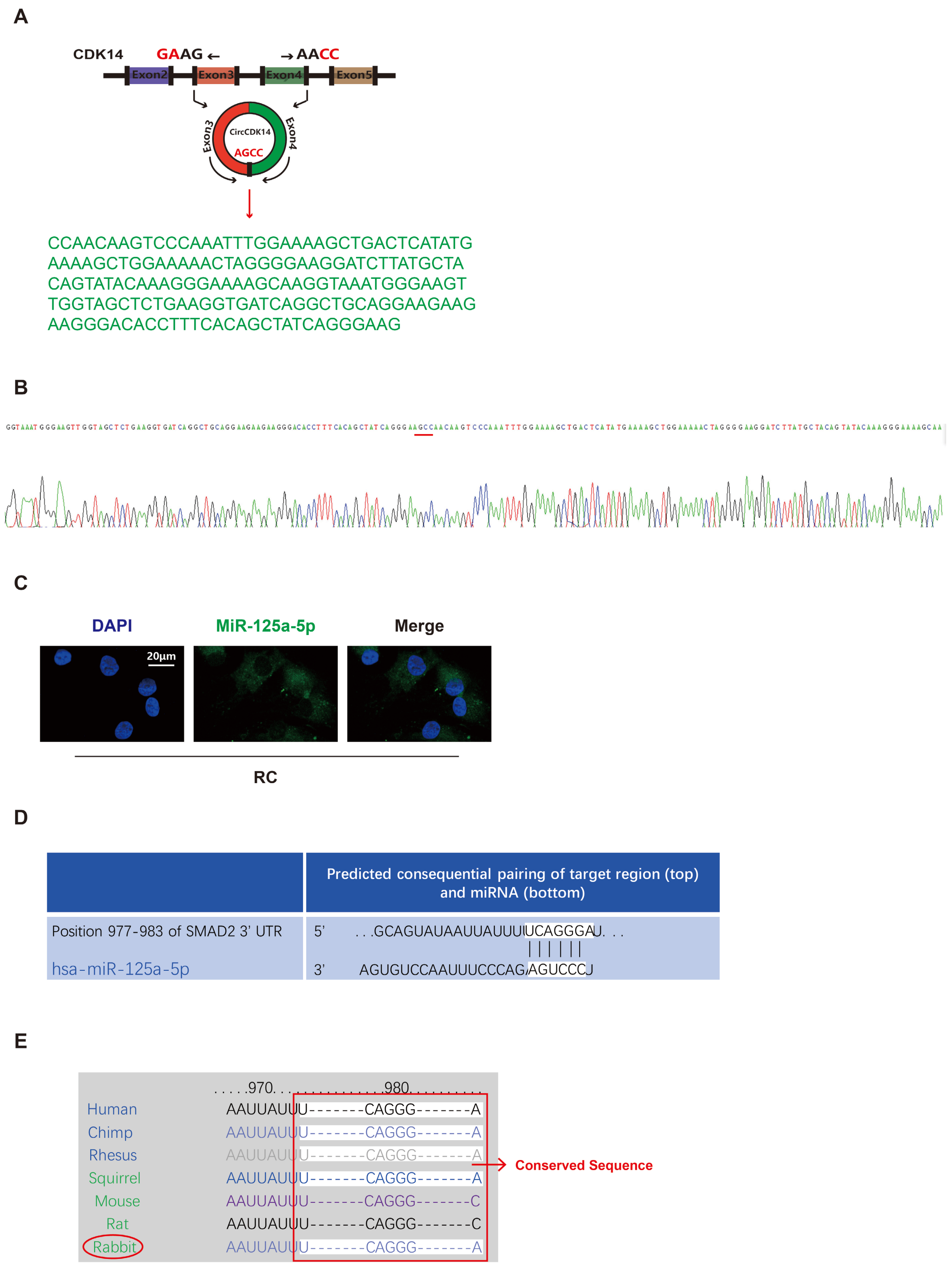

Figure S2 (A) The whole sequence of CircCDK14 provided by Circbase database. **(B)** The whole sequence of CircCDK14 detected by Sanger sequencing. **(C)** RNA FISH showed that the probe of human miR-125a-5p could also bind to miR-125a-5p in rabbit chondrocytes. Scale bar, 20μm **(D)** Putative miR-125a-5p binding site in the 3′UTR of Smad2 mRNA. **(E)** Sequence alignment of a putative miR-125a-5p binding site within the 3′UTR of Smad2 mRNA shows a high level of sequence conservation and complementarity with miR-125a-5p.

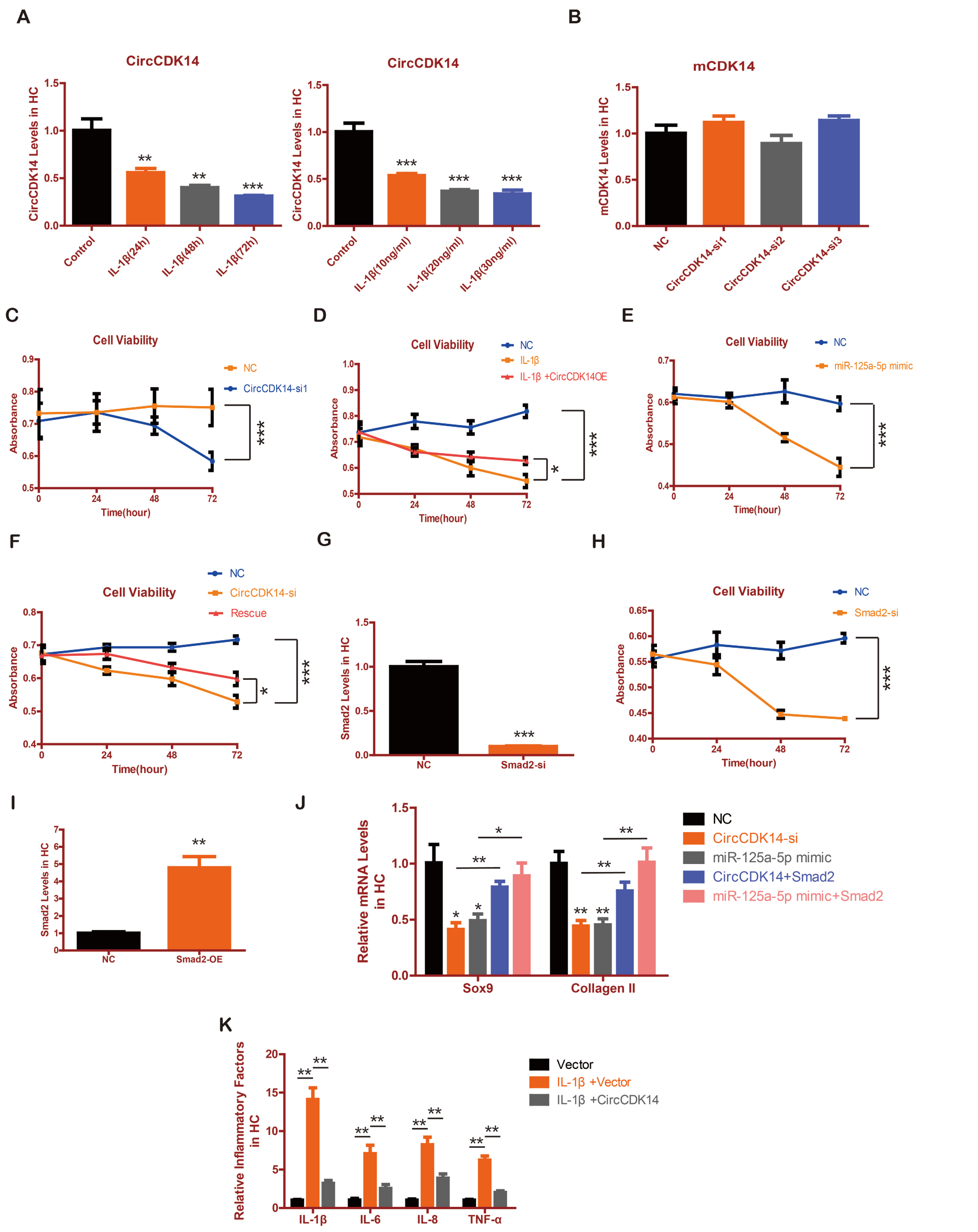

Figure S3 (A) Expression of CircCDK14 after IL-1β (10ng/mL) treatment with different time gradient. Expression of CircCDK14 after 24 h IL-1β treatment with different concentration gradient. (n=3) **p<0.01, ***p<0.001. **(B)** Three siRNAs specific against CircCDK14 have no effect on CDK14 mRNA. **(C)** CCK8 assay demonstrated that siRNA-mediated CircCDK14 knockdown suppressed HC cell proliferation. Data represents the mean ± SD (n = 5). ***p<0.001. **(D)** CCK8 assay demonstrated that the treatment of IL-1β suppressed HC cells proliferation, while the overexpression of CircCDK14 could antagonize this effect. Data represents the mean ± SD (n = 5). *p<0.05, ***p<0.001. **(E)** CCK8 assay demonstrated that the overexpression of miR-125a-5p suppressed HC cell proliferation. Data represents the mean ± SD (n = 5). ***p<0.001. **(F)** CCK8 assay showed the effect of siRNA-mediated CircCDK14 knocking down on HC cell proliferation could be rescued by inhibition of miR-125a-5p. *p<0.05, ***p<0.001. **(G)** The knock down efficiency of Smad2 in HC was detected by qRT-PCR. (n=3) ***p<0.001.

(H) CCK8 assay demonstrated that siRNA-mediated Smad2 knockdown suppressed HC cell proliferation. Data represents the mean ± SD (n = 5). ***p<0.001. **(I)** The overexpression efficiency of Smad2 in HC detected by qRT-PCR. (n=3) **p<0.01. **(J)** qRT-PCR showed that overexpression of Smad2 could antagonize the effects of CircCDK14-si and miR-125a-5p mimic on Sox9 and Collagen II. (n=3) *p<0.05, **p<0.01. **(K)** qRT-PCR showed that the overexpression of CircCDK14 inhibited IL-1β-induced inflammatory response in HC. (n=3) **p<0.01.

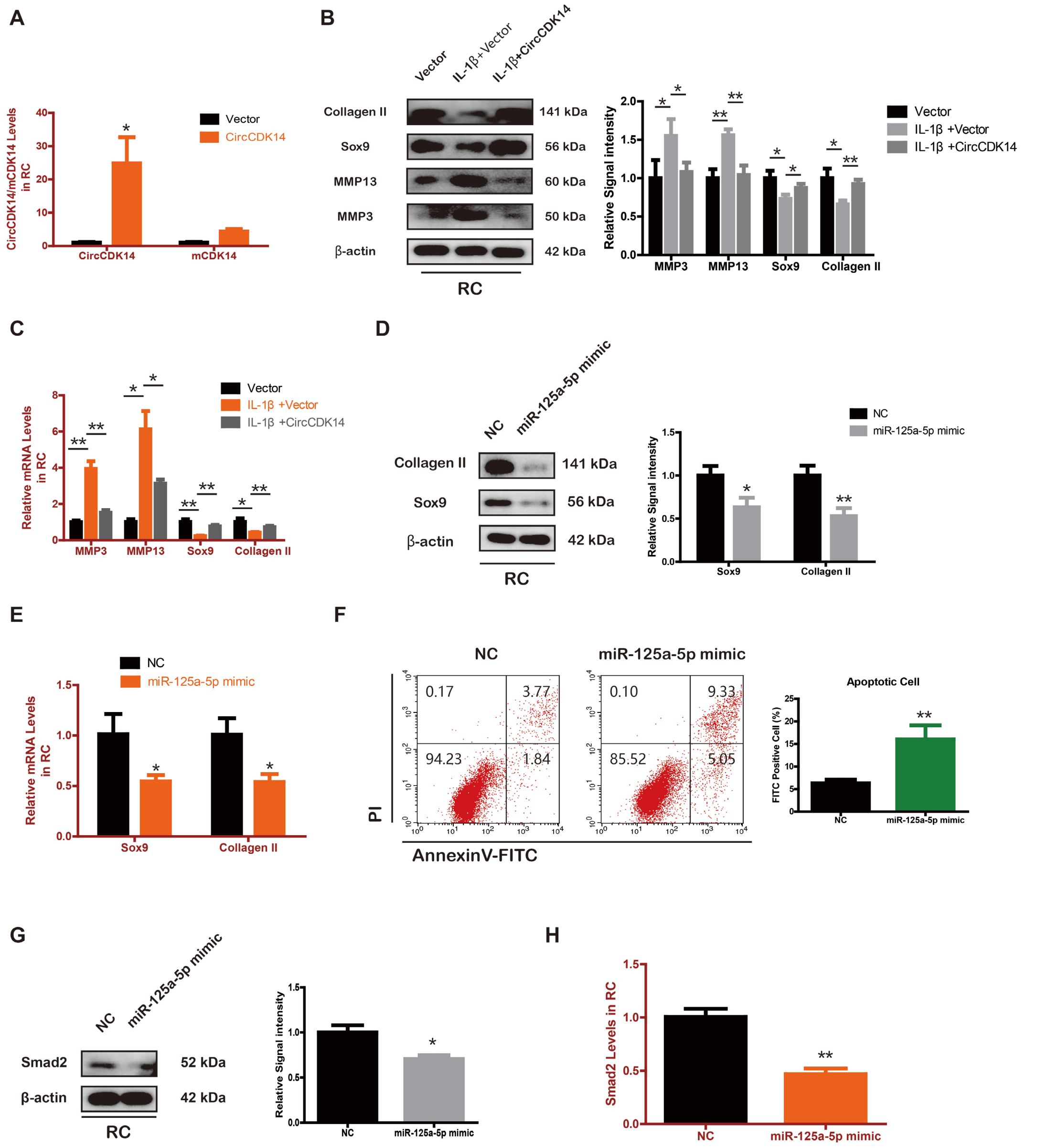

Figure S4 (A) The overexpression efficiency of CircCDK14 in RCs detected by qRT-PCR. (n=3) *p<0.05. **(B)** Western blot analysis of MMP3, MMP13, Sox9 and Collagen II in RCs after treating with IL-1β (10ng/mL) for 24 h and the saving effects of overexpressed CircCDK14 on IL-1β. The optical density analysis was performed from the results of three independent experiments of Western blot samples. *p<0.05, **p<0.01. **(C)** The expression levels of MMP3, MMP13, Sox9 and Collagen II in RCs was detected by qRT-PCR after treating with IL-1β (10ng/mL) for 24 h and the saving effects of overexpressed CircCDK14 on IL-1β. (n=3) *p<0.05, **p<0.01. **(D)** Western blot analysis of Sox9 and Collagen II in RCs when miR-125a-5p was upregulated. The optical density analysis was performed

from the results of three independent experiments of Western blot samples. *p<0.05, **p<0.01. **(E)** The expression levels of Sox9 and Collagen II in RCs were detected by qRT-PCR when miR-125a-5p was upregulated. (n=3) *p<0.05. **(F)** RCs were transfected with miR-125a-5p mimic, followed by Flow cytometry assay. The percentage of apoptotic cells was shown. (n=3) **p<0.01. **(G)** Western blot analysis of Smad2 in RCs when treated with miR-125a-5p mimic. The optical density analysis was performed from the results of three independent experiments of Western blot samples. *p<0.05. **(H)** The expression level of Smad2 in RCs was detected by qRT-PCR when miR-125a-5p was upregulated. (n=3) **p<0.01.

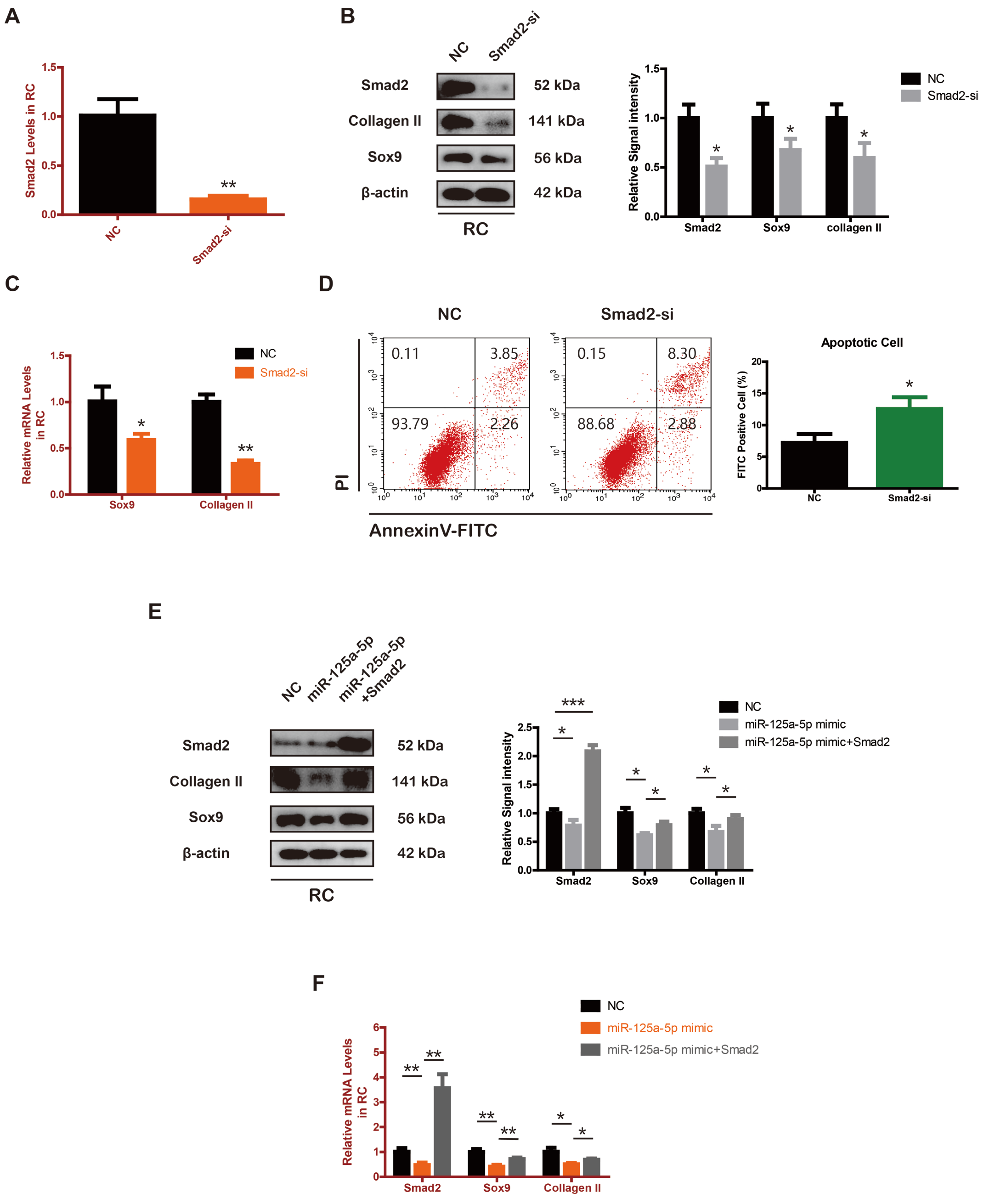

Figure S5 (A) The knock down efficiency of Smad2 in RCs was detected by qRT-PCR. (n=3) **p<0.01. **(B)** Western blot analysis of Smad2, Sox9 and Collagen II when Smad2 was downregulated in RCs. The optical density analysis was performed from the results of three independent experiments of Western blot samples. *p<0.05. **(C)** The expression level of Sox9 and Collagen II in RCs was detected by qRT-PCR

when treated with Smad2-si. (n=3) *p<0.05, **p<0.01. **(D)** Flow cytometry experiment indicated that siRNA-mediated Smad2 knockdown increased RCs apoptosis rate. (n=3) *p<0.05. (**E**) Western blot analysis of Smad2, Sox9 and Collagen II when Smad2 was upregulated in RCs. The optical density analysis was performed from the results of three independent experiments of Western blot samples. *p<0.05. ***p<0.001. (**F**) The expression level of Smad2, Sox9 and Collagen II in RCs was detected by qRT-PCR when Smad2 was upregulated in RCs. (n=3) *p<0.05, **p<0.01.

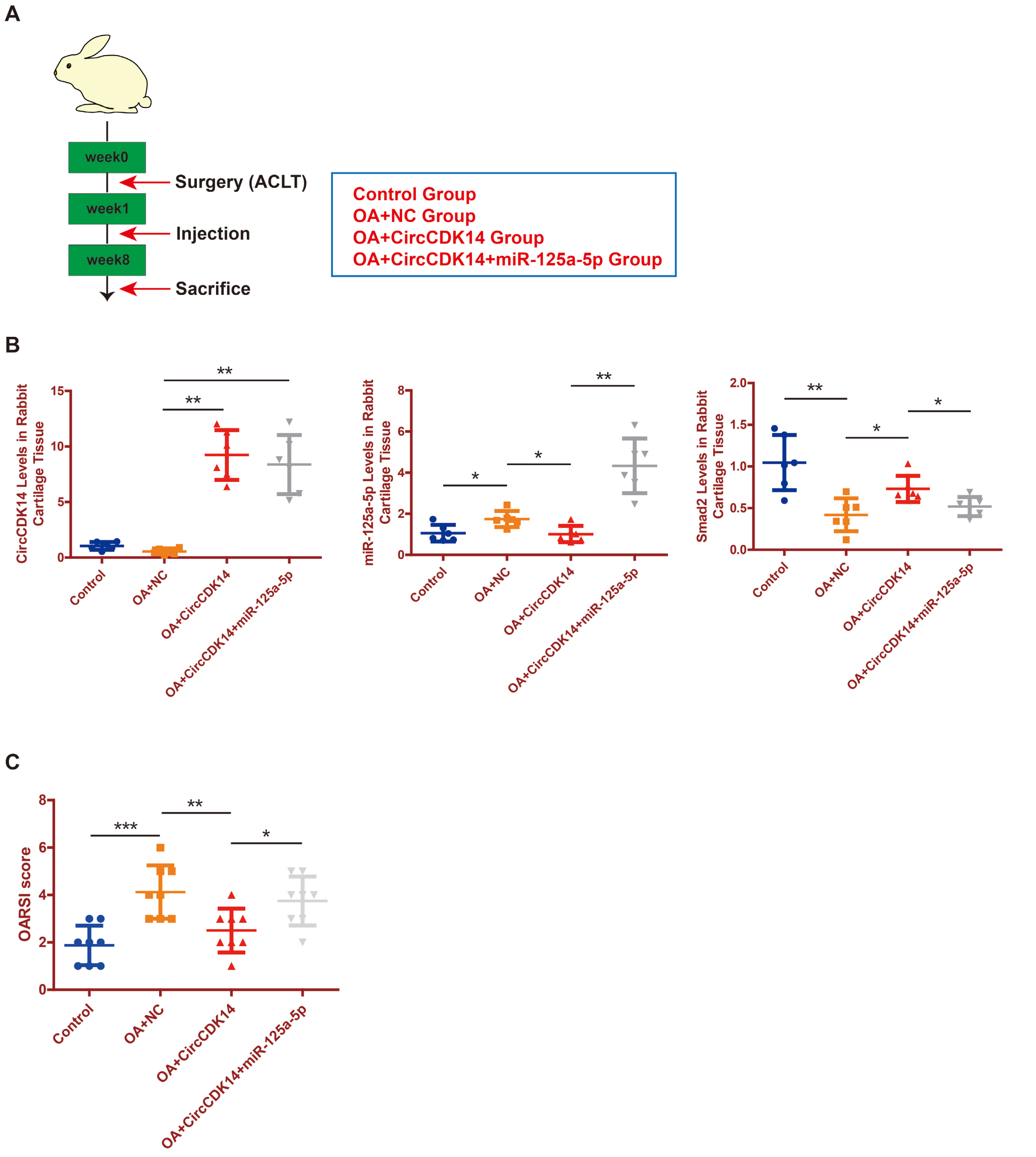

Figure S6 (A) Establishment of a rescue model for rabbit OA targeting CircCDK14. **(B)** The expression levels of CircCDK14, miR-125a-5p and Smad2 in rabbit joint tissues were detected by qRT-PCR (n=6) *p<0.05. **p<0.01. (**C**) OARSI scoring was performed according to Safranin-O/fast green staining results. (n=8) *p<0.05, **p<0.01.

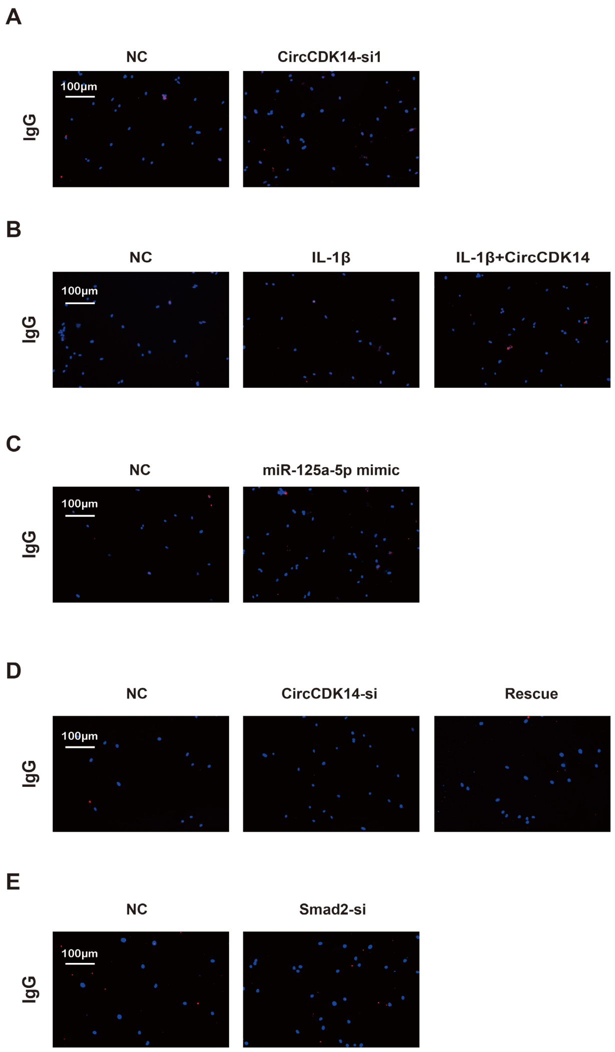

Figure S7 (A) Negative control (IgG) of IF in NC and CircCDK14-si1 groups. scale bar, 100μm. (**B**) Negative control (IgG) of IF in NC, IL-1β and IL-1β+CircCDK14 groups. scale bar, 100μm. (**C**) Negative control (IgG) of IF in NC and miR-125a-5p groups. scale bar, 100μm. (**D**) Negative control (IgG) of IF in NC, CircCDK14-si and rescue groups. scale bar, 100μm. (**E**) Negative control (IgG) of IF in NC and Smad2-si groups. scale bar, 100μm.

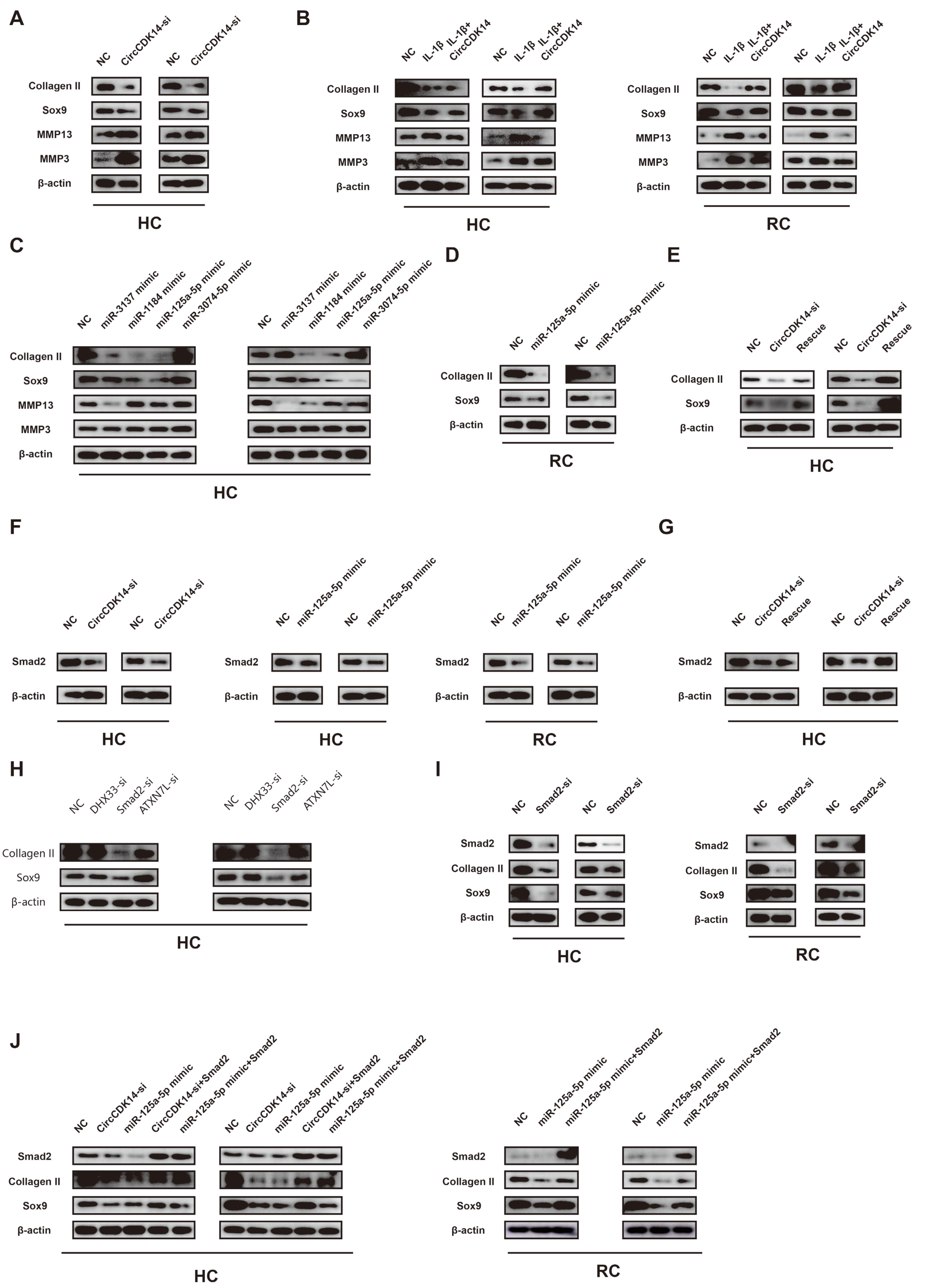

Figure S8 (A) Western blots of MMP3, MMP13, Sox9 and Collagen II when CircCDK14 was downregulated in HCs. (**B**) Western blots of MMP3, MMP13, Sox9 and Collagen II in HCs and RCs after treating with IL-1β (10ng/mL) for 24 h and the saving effects of overexpressed CircCDK14 on IL-1β. (**C**) Western blots of MMP3, MMP13, Sox9 and Collagen II in HCs when the selected 4 candidate miRNAs were overexpressed respectively. (**D**) Western blots of Sox9 and Collagen II in RCs when miR-125a-5p was upregulated. (**E**) Western blots showed that the downregulation of miR-125a-5p antagonized the effect of CircCDK14-si on Sox9 and Collagen II in HCs. (**F**) Western blots of Smad2 when treated with CircCDK14-si or miR-125a-5p mimic in HCs and RCs. (**G**) Western blots showed that the

downregulation of miR-125a-5p antagonized the effect of CircCDK14-si on Smad2 in HCs. (**H**) Western blots of Sox9 and Collagen II when the selected 3 candidate mRNAs were downregulated respectively in HCs. (**I**) Western blots of Smad2, Sox9 and Collagen II when Smad2 was downregulated in HCs and RCs. (**J**) Western blots of Smad2, Sox9 and Collagen II when Smad2 was upregulated in HCs and RCs.

Tables

Table S1 Primer sequences used in this study

Primer set	Gene ID	Primers	Sequences
CircCDK14<br>(human, Divergent)		Foward<br>Reverse	TGGGAAGTTGGTAGCTCTGAAG<br>AGATCCTTCCCCTAGTTTTTCC
mCDK14<br>(human, Convergent)		Foward<br>Reverse	GCTGGAAAAACTAGGGGAAGG<br>CTTCCTGCAGCCTGATCACC
β-actin<br>(human)	60	Foward<br>Reverse	AGAGCTACGAGCTGCCTGAC<br>AGCACTGTGTTGGCGTACAG
GAPDH<br>(human)	2597	Foward<br>Reverse	AGCCACATCGCTCAGACAC<br>GCCCAATACGACCAAATCC
MMP3<br>(human)	4314	Foward<br>Reverse	CCTACAAGGAGGCAGGCAAG<br>CCCGTCACCTCCAATCCAAG
MMP13<br>(human)	4322	Foward<br>Reverse	TCGGCCACTCCTTAGGTCTT<br>AAGTGGCTTTTGCCGGTGTA
Sox9<br>(human)	6662	Foward<br>Reverse	GCTCTGGAGACTTCTGAACGA<br>CCGTTCTTCACCGACTTCCT
Collagen II<br>(human)	1280	Foward<br>Reverse	CCAGATGACCTTCCTACGCC<br>TTCAGGGCAGTGTACGTGAAC
Smad2<br>(human)	4087	Foward<br>Reverse	GGCGGAGAAGCAGCTCG<br>ACTGGAGGCAAAACTGGTGT
IL-6<br>(human)	3569	Foward<br>Reverse	ACTCACCTCTTCAGAACGAATTG<br>CCATCTTTGGAAGGTTCAGGTTG
IL-8<br>(human)	3576	Foward<br>Reverse	TTTTGCCAAGGAGTGCTAAAGA<br>AACCCTCTGCACCCAGTTTTC
TNF-α<br>(human)	7124	Foward<br>Reverse	CCTCTCTCTAATCAGCCCTCTG<br>GAGGACCTGGGAGTAGATGAG
IL-1β<br>(human)	3553	Foward<br>Reverse	ATGATGGCTTATTACAGTGGCAA<br>GTCGGAGATTCGTAGCTGGA
KLF13<br>(human)	51621	Foward<br>Reverse	CGGCCTCAGACAAAGGGTC<br>TTCCCGTAAACTTTCTCGCAG
MAP2K7<br>(human)	5609	Foward<br>Reverse	CCACGTCATTGCCGTTAAGC<br>GCACGATGTAGGGGCAGTC
LIN28B<br>(human)	389421	Foward<br>Reverse	CATCTCCATGATAAACCGAGAGG<br>GTTACCCGTATTGACTCAAGGC
ARID3A<br>(human)	1820	Foward<br>Reverse	ACCACGGCGACTGGACTTA<br>CACAGGTGTCCCTCGCTTC
DHX33<br>(human)	56919	Foward<br>Reverse	GATGAAGCTCACGAACGGACT<br>CCACATCCATCGTAGCTGACA
ZSWIM6<br>(human)	57688	Foward<br>Reverse	AAGCGGCTGCGTAGACAAC<br>GGCTCCGATTGTATTGCAGGT
HK2	3099	Foward	GAGCCACCACTCACCCTACT

(human)		Reverse	CCAGGCATTCGGCAATGTG
MAP3K1<br>(human)	4214	Foward<br>Reverse	CATCAGGTCGCACAGTGAAAT<br>TCAGGGCTATATGGTGAGAAGC
LIFR<br>(human)	3977	Foward<br>Reverse	TGGAACGACAGGGGTTCAGT<br>GAGTTGTGTTGTGGGTCACTAA
ZDHHC9<br>(human)	51114	Foward<br>Reverse	CCCAGGCAGGAACACCTTTT<br>CCGAGGAATCACTCCAGGG
ATXN7L3<br>(human)	56970	Foward<br>Reverse	ACAGCAAACTAGAGGCCATCG<br>CCTTCATGCTATCAGGGTCCG
GAPDH<br>(Rabbit)	100009074	Foward<br>Reverse	GCTTCTTCTCGTGCAGTGCTA<br>GATGGCCTTCCCGTTGATGA
MMP3<br>(Rabbit)	100009111	Foward<br>Reverse	CAATCCCTCTATGGACCGGC<br>CAGCAGCCTGTTTGAACACC
MMP13<br>(Rabbit)	100008685	Foward<br>Reverse	CCAGTCTCTCTATGGTCCAGG<br>TGCTTCCGCATAGCACTGAA
Sox9<br>(Rabbit)	100008944	Foward<br>Reverse	GGGGTGATGAGCTTTGCTGA<br>AAAACAGGATCTGTCGCCGT
Collagen II<br>(Rabbit)	100009005	Foward<br>Reverse	GGATAGACCCCAACCAAGGC<br>GGGTCCTTTGGCTGTTCAGA
Smad2<br>(Rabbit)	100359137	Foward<br>Reverse	GCTCTTTTCGCAGTGTGGTC<br>CACCTGAAGACGACCGTCAA
U6<br>(human)	26827	Foward<br>Reverse	CTCGCTTCGGCAGCACA<br>AACGCTTCACGAATTTGCGT
hsa-miR-211-5p	406993	Foward<br>Reverse	GTTCCCTTTGTCATCCTTCGCCT<br>Universal Reverse Primer (CWBIO)
hsa-miR-5088-3p	100847074	Foward<br>Reverse	TCCCTTCTTCCTGGGCCCTCA<br>Universal Reverse Primer (CWBIO)
hsa-miR-6875-3p	102466755	Foward<br>Reverse	CCATTCTTCCTGCCCTGGCTCCAT<br>Universal Reverse Primer (CWBIO)
hsa-miR-3137	100422926	Foward<br>Reverse	TATTCTGTAGCCTGGGAGCAATGGG<br>Universal Reverse Primer (CWBIO)
hsa-miR-4778-3p	100616464	Foward<br>Reverse	CGCGCTCTTCTTCCTTTGCAGAGTTGA<br>Universal Reverse Primer (CWBIO)
hsa-miR-204-5p	406987	Foward<br>Reverse	CGTTCCCTTTGTCATCCTATGCCT<br>Universal Reverse Primer (CWBIO)
hsa-miR-1184	100302111	Foward<br>Reverse	CCTGCAGCGACTTGATGGC<br>Universal Reverse Primer (CWBIO)
hsa-miR-937-5p	100126338	Foward<br>Reverse	GTGAGTCAGGGTGGGGCT<br>Universal Reverse Primer (CWBIO)
hsa-miR-125a-5p	406910	Foward<br>Reverse	TCCCTGAGACCCTTTAACCTGTGA<br>Universal Reverse Primer (CWBIO)
hsa-miR-3667-3p	100500882	Foward<br>Reverse	AGACCTTCCTCTCCATGGGTCTTT<br>Universal Reverse Primer (CWBIO)

hsa-miR-7156-5p	102466995	Foward<br>Reverse	CTGCAGCCACTTGGGGAACTGGT<br>Universal Reverse Primer (CWBIO)
hsa-miR-3074-5p	100422842	Foward<br>Reverse	AGTTCCTGCTGAACTGAGCCAG<br>Universal Reverse Primer (CWBIO)

Table S2 Details about type of OARSI scoring (mean/max/sum)

	Crotrol	OA+NC	OA+CircCDK14	OA+CircCDK14+miR-125a-5p
Mean	1.88	4.13	2.50	3.75
Max	3	6	4	5
Min	1	2	1	2
Sum	15	33	20	30
Stdev	0.78	1.05	0.87	0.97

Table S3 Sequences of siRNAs, miRNA mimic/inhibitor and probes

CircCDK14 siRNA#1	GAAGCCAACAAGTCCCAAA
CircCDK14 siRNA#2	TATCAGGGAAGCCAACAAG
CircCDK14 siRNA#3	CAGGGAAGCCAACAAGTCC
Smad2 siRNA	GAATTGAGCCACAGAGTAA
DHX33 siRNA	GTGTGATGCTTCAGCTTCT
ATXN7L3 siRNA	GGCCTAGGTTCCAACAAGA
miR-125a-5p mimic	RiboBio
miR-125a-5p inhibitor	RiboBio
miR-3137 mimic	RiboBio
miR-1184 mimic	RiboBio
miR-3074-5p mimic	RiboBio
Probes for FISH	
CircCDK14	5′-CY3-GGACTTGTTGGCTTCCCTGATAGCTG-3′
MiR-125a-5p	5′-FAM-TCACAGGTTAAAGGGTCTCAGGGA-3′
Probes for Pull-down assay	
CircCDK14	CTTGTTGGCTTCCCTGATAG

Table S4 Maps of vectors used in this study

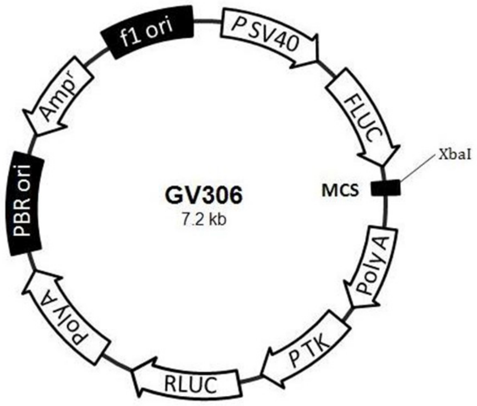

Firefly_Luciferase-Renilla_Luciferase vector

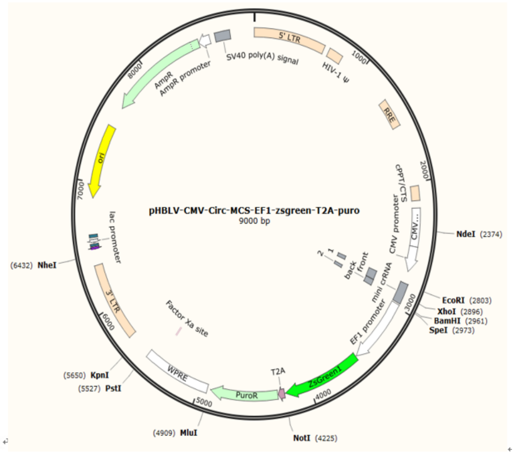

pHBLV-CMV-Cicr-MCS-EF1-zsgreen-t2a-puro vector

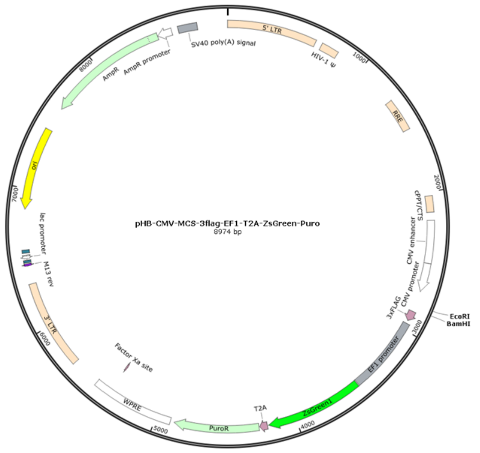

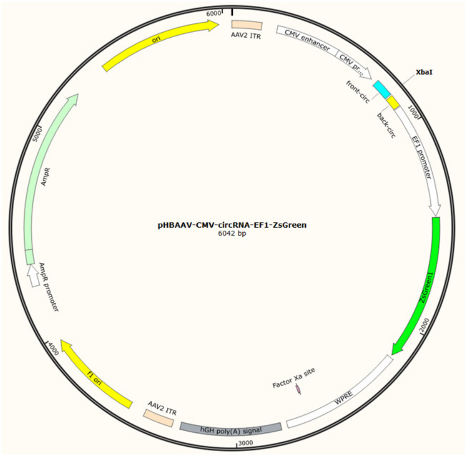

pHB-CMV-MCS-EF1-zsgreen-t2a-puro

pHBAAV-CMV-MCS-3flag-EF1-ZsGreen vector

Table S5 RNAseq Analyses

id	Circbase id	log2FC(OA/Control)	pval	Fdr	regulate	significant
17:27765535|277665881	hsa_circ_0106434	-7.2095933	2.94E-10	1.78E-06	down	yes
1:102979382|102989571	hsa_circ_0110251	-7.0146886	9.33E-11	1.13E-06	down	yes
19:6702127|6702579	hsa_circ_0002130	-5.7256104	3.17E-08	0.00012766	down	yes
8:17558295|17562110	hsa_circ_0005805	-5.830035	7.60E-07	0.00183471	down	yes
5:59180595|59215968	hsa_circ_0072568	-5.2760784	1.18E-07	0.00035593	down	yes
5:65170617|65215487	hsa_circ_0008667	-3.9400185	6.71E-05	0.06130968	down	yes
4:88475841|88479507	hsa_circ_0070421	-4.5431287	4.12E-06	0.00710284	down	yes
7:90747681|90790652	hsa_circ_0001722	-4.4750758	3.16E-06	0.00636129	down	yes
4:150467673|150491035	hsa_circ_0006867	-4.2555494	1.27E-05	0.01510205	down	yes
17:27778690|27779056	None	-4.2259783	1.16E-05	0.01510205	down	yes
2:223912253|223918010	hsa_circ_0008365	-4.0531467	0.00013432	0.0878503	down	yes
20:54157169|54171670	hsa_circ_0060927	-4.2436025	8.27E-05	0.06130968	down	yes
9:6880012|6893232	hsa_circ_0001839	4.487324826	0.333264016	1	down	yes
5:65170617|65215487	hsa_circ_0008667	-3.9400185	6.71E-05	0.06130968	down	yes

1:46077515 | 46080750	hsa_circ_0003161	-3.9505609	8.63E-05	0.06130968	down	yes
8:17543318 | 17543715	hsa_circ_0083429	-4.576294	1.42E-05	0.01510205	down	yes
15:83923904 | 83970637	hsa_circ_0104766	-3.7749837	0.0001382	0.0878503	down	yes
19:6697344 | 6697794	None	-3.5244413	0.00030733	0.17675827	down	yes
20:45815541 | 45815913	hsa_circ_0060551	-3.6184204	0.00038076	0.1999479	down	yes
1:27668226 | 27669346	hsa_circ_0002470	-3.85047	0.00106467	0.38966852	down	yes
7:84110470 | 84134951	hsa_circ_0134888	-3.4826538	0.00296286	0.73284844	down	yes
12:100204940 | 100205744	hsa_circ_0027857	-3.4815857	0.0009696	0.37359312	down	yes
19:48845370 | 48852326	hsa_circ_0051782	-3.44724	0.00059645	0.2705299	down	yes
21:41432050 | 41437152	None	-3.5076824	0.00084407	0.36409636	down	yes
4:102304317 | 102315830	hsa_circ_0002782	-3.8014826	0.00336046	0.73805944	down	yes
1:93305388 | 93325895	None	-3.4219483	0.00098981	0.37359312	down	yes
5:72861808 | 72865729	hsa_circ_0002692	0.599662703	0.482010652	1	down	yes
12:100204940 | 100205757	hsa_circ_0096948	-3.3785056	0.00159788	0.47739629	down	yes
3:29868858 | 29899755	hsa_circ_0064644	1.782047111	0.157236939	1	down	yes
5:138424075 | 138425582	hsa_circ_0004763	-3.355593	0.00142197	0.4519615	down	yes
19:41248514 | 41248820	hsa_circ_0002882	-1.376539581	0.205755968	1	down	yes
13:75713182 | 75727098	hsa_circ_0030441	4.06243391	4.95E-06	0.00748029	up	yes
10:115120185 | 115129535	hsa_circ_0020093	3.52956202	1.50E-05	0.01510205	up	yes
8:41661430 | 41661941	hsa_circ_0001792	3.31749642	7.68E-05	0.06130968	up	yes
1:247159006 | 247159813	hsa_circ_0017348	5.42175065	8.25E-05	0.06130968	up	yes
1:246591512 | 246591941	hsa_circ_0017310	3.25903817	0.00043672	0.21098767	up	yes

11:78119182 | 78121174	hsa_circ_0008019	3.15142761	0.00315668	0.73805944	up	yes
11:63895159 | 63895633	hsa_circ_0022614	2.51804111	0.00221361	0.58121793	up	yes
2:72674532 | 72733118	None	4.56218595	0.00098611	0.37359312	up	yes
8:30474779 | 30479377	hsa_circ_0007409	-0.955555813	0.652030171	1	up	yes
9:84702159 | 84710791	hsa_circ_0139142	2.86567306	0.00336093	0.73805944	up	yes
7:157366502 | 157367483	hsa_circ_0002451	2.1840816	0.01295983	1	up	yes
10:32805015 | 32824604	hsa_circ_0093573	2.47034385	0.00779452	1	up	yes
9:120536372 | 120539164	None	2.2484411	0.00992009	1	up	yes
9:87698656 | 87700237	hsa_circ_0087421	-2.095067331	0.068295109	1	up	yes
20:38040435 | 38066256	hsa_circ_0008006	2.91024396	0.00929394	1	up	yes
11:92352096 | 92355404	hsa_circ_0000348	4.16268873	0.00303382	0.73284844	up	yes
21:36338779 | 36344707	None	4.11644082	0.00199654	0.54804915	up	yes
20:25496663 | 25498346	hsa_circ_0004812	2.04618052	0.00300782	0.73284844	up	yes
16:58560212 | 58560362	None	3.91773309	0.00679791	1	up	yes
5:32724698 | 32739030	hsa_circ_0072107	2.31448473	0.01628771	1	up	yes
6:168543625 | 168608239	hsa_circ_0078696	4.56983277	0.00170875	0.4799612	up	yes
2:241030284 | 241030571	hsa_circ_0004526	2.3421537	0.00954736	1	up	yes
20:17956933 | 17961326	hsa_circ_0009173	-2.010872082	0.096621893	1	up	yes
3:111913319 | 111920419	hsa_circ_0001326	-1.042522917	0.326337728	1	up	yes
1:23071225 | 23072197	hsa_circ_0010835	3.52357915	0.00111472	0.39598924	up	yes
20:64022757 | 64026058	hsa_circ_0115583	2.15899955	0.01569604	1	up	yes
5:72877228 | 72883232	None	2.0345252	0.01078439	1	up	yes

4:105691976|105695668	hsa_circ_0007668	2.60053265	0.022674 81	1	up	yes
